# Spatial maps of hepatocellular carcinoma transcriptomes reveal spatial expression patterns in tumor immune microenvironment

**DOI:** 10.7150/thno.71873

**Published:** 2022-05-16

**Authors:** Yue-fan Wang, Sheng-xian Yuan, Hui Jiang, Zhi-xuan Li, Hao-zan Yin, Jian Tan, Zhi-hui Dai, Chun-mei Ge, Shu-han Sun, Fu Yang

**Affiliations:** 1 The department of Medical Genetics, Naval Medical University, Shanghai, 200433, China.; 2 The Third Department of Hepatic Surgery, Eastern Hepatobiliary Surgery Hospital Affiliated to Naval Medical University, Shanghai, 200438, China.; 3 Department of Pathology, Changhai Hospital Affiliated to Navy Medical University, Shanghai, 200433, China.; 4 Translational Medicine Research Center, Medical Innovation Research Division and Fourth Medical Center of the Chinese PLA General Hospital, Beijing, 100048, China.

**Keywords:** hepatocellular carcinoma, spatial transcriptomics, heterogeneity, immune microenvironment

## Abstract

**Rationale:** Hepatocellular carcinoma (HCC) is a highly heterogeneous and malignant disease with the complex immune microenvironment, which ultimately influence clinic outcomes of patients. However, the spatial expression patterns of diverse immune cells among tumor microenvironment remain to be further deciphered.

**Methods:** Spatial transcriptomics sequencing (ST) was implemented on two portions of HCC specimens. Differentially expressed genes, cell cycle phases, epithelial-mesenchymal features, pseudo-time and immune infiltration analysis were applied to demonstrate the intratumor heterogeneity and define the specific immune-related regions, and the results were further validated by a second analysis on another ST study. *In vitro* and *in vivo* experiments were conducted to confirm the functional mechanisms of key molecules such as CCL15, CCL19 and CCL21. Clinical tissue samples were used to assess their potential prognostic and therapeutic values.

**Results:** Totally, 7553 spots were categorized into 15 subsets by hierarchical clustering, and malignant subsets with intratumor heterogeneity phenotypes were identified. Spatial heterogeneity from distinct sectors highlights specific chemokines: CCL15 is remarkable in the core region of the carcinoma sector and facilitates the immunosuppressive microenvironment by recruiting and polarizing M2-like macrophages *in vitro* and *in vivo*; High expression of CCL15 and CD163 respectively predicts poor prognosis of HCC patients, and the combined application of them has better predictive value. CCL19 and CCL21, sharing similar spatial expression patterns, are highly-correlated and prominent in the immune infiltration enrichment and recruit CD3^+^ T cells and CD20^+^ B cells to inhibit the growth of HCC, indicating a good prognosis of HCC patients.

**Conclusions:** Taken together, our studies preliminarily reveal intratumor heterogeneity of HCC based on ST techniques and unravel the previously unexplored spatial expression patterns in the immune microenvironment. We also highlight the clinical significance and spatial discrepancy of key molecules, providing novel insight for further developing therapeutic strategies in HCC.

## Introduction

The latest cancer statistics for 2020 indicate that primary liver cancer (PLC) ranks sixth in morbidity and third in mortality worldwide, with approximately 906,000 new cases and 830,000 deaths yearly, and that hepatocellular carcinoma (HCC) accounts for approximately 80% of PLC cases 1. Vaccination against hepatitis B virus (HBV) and reduction in aflatoxin exposure have sharply decreased the incidence of HCC in recent decades 2. HCC screening and early diagnosis, as well as advances in surgery and molecular targeted medicine, have dramatically reduced the mortality rate of HCC to a certain extent. Nonetheless, there still exist clinical phenomena in which although the clinicopathological stages of some patients are consistent, major discrepancies due to tumor heterogeneity are noted regarding drug sensitivity and prognosis after operation 3.

Tumor heterogeneity in HCC can be roughly classified into phenotypic heterogeneity and molecular heterogeneity 4, with the former being observed from pathological and morphological perspectives and the latter being documented at three levels: interpatient, intertumoral, and intratumor heterogeneity 5, 6. Previous studies have attempted to profile the landscape of molecular heterogeneity in HCC 7-9; nevertheless, due to discrepancies, the roles of various factors in liver carcinogenesis and progression remain ill-defined. Accumulating evidence has found that tumor heterogeneity is closely associated with the immune environment, which is characterized by three immunosubtypes (immune-high, immune-mid, and immune-low) with noteworthily distinct prognoses 10. Notably, single-cell RNA sequencing (scRNA-seq) has dramatically enriched our knowledge of tumor heterogeneity in tumor cell subpopulations, the immune microenvironment, and the cell developmental trajectory of HCC 11-13. Whereas spatial information is disrupted after tissue dissection into a single-cell suspension, disengaging gene expression from its original tissue architecture and rendering exploration of the authentic appearance of gene expression patterns difficult 14. In this scenario, emerging spatial transcriptomics (ST) technology has the attributes of spatially localizing function resulting from gene expression, which can be a perfect complement to scRNA sequencing. ST was first applied in the mouse brain and human breast cancer to verify the feasibility of spatial visualization and quantitative analysis of gene expression 15; it was then used to explore the spatial discrepancy of gene expression in dynamic disease processes, such as amyotrophic lateral sclerosis 16 and Alzheimer's disease 17, as well as in normal tissue development processes 18. Moreover, ST is conducive to resolving spatial heterogeneity and spatial gene expression patterns in various tumors, such as prostate cancer 19, melanoma 20 and pancreatic ductal adenocarcinoma 21, and primary liver cancer 22, 23.

In the present study, we performed ST sequencing for two portions of HCC specimens with clearly demarcated and continuous cores, peripheral regions, and boundary regions and investigated the potential makers and regulatory mechanisms of immunosuppressive or immune enrichment regions with distinct prognoses based on analyzing spatial gene expression patterns. Accordingly, our studies highlighted the spatial heterogeneity of gene expression patterns as well as their clinical significance in the tumor immune microenvironment and provided novel insight for the further prognostic and therapeutic strategies for HCC.

## Materials and Methods

### Specimens and clinical data

Totally, two fresh continuous tissues (from tumor tissue, tumor boundary region to para-tumor tissue) of one HCC case were acquired from surgical resections without preoperative treatment at Eastern Hepatobiliary Surgery Hospital (EHBH, Shanghai, China). Another 89 HCC tissues were obtained from the Department of Pathology at Changhai Hospital (CH, Shanghai, China). Diagnoses of HCC were evaluated by two certificated pathologists. Collections of tissue specimens were approved by the Ethics Committee of EHBH and CH, respectively. Written informed consent was provided by individuals donating HCC tissues. Fresh tissues for ST sequencing were promptly filled with optimal cutting temperature (OCT) compound and then snap frozen in isopentane and liquid nitrogen. The samples used for Western blotting were freshly frozen in -80 °C until use. Tissues utilized for immunohistochemistry analysis were formalin fixed and paraffin embedded (FFPE). Clinical characteristics from CH cohort were listed in Table S1.

### Spatial transcriptomics sequencing and bioinformatic analysis

#### Spatial transcriptomics sequencing

The two fresh HCC tissues were surgically resected, washed with pre-cooled PBS solution and drained by gauzes. The tissues were then transferred to isopentane for soaking and freezing, and moved to a cryopreservation tube with tweezers for subsequent embedding with the OCT mixture. The procedures are as follows: (1) Hematoxylin-eosin staining (HE) was used for histological imaging; (2) Tissues were then fixed, stained and permeabilized to release mRNA, which can bind to the probes that contained a 16 bp spot barcode and a 12 bp UMI sequence. The capturing probe with poly (dT) sequence acquired gene expression information by binding to mRNA 3'-poly(A) tail; (3) cDNA synthesis and sequencing libraries were prepared using the captured RNA as templates; (4) This sequencing was based on 10× Genomics Visium and the paired-end sequencing mode of the Illumina sequencing platform, which was previously described before 24.

#### Data processing and quality control

After sequencing, the data were visualized and analyzed via Space Ranger (version1.1.0). The Space Ranger was used to ensemble the reference genome database and FastQC software was utilized for data quality control. Further sequencing and application of R software and other applications were used for data visualization.

R software version 4.0.3 and “Seurat” software package version 3.1.1 were used for analysis. After excluding low-quality units, we used the “SCTransform” function to normalize data, find variable features and scale data.

#### Dimensionality reduction analysis

Next, principal component analysis (PCA) was performed on the first 2000 highly variable genes using the “RunPCA” function. Then, the number of PCs corresponding to ElbowPlot was selected and the “RunUMAP” function with a default perplexity value of 30 was executed to obtain the bidimensional coordinates for single-spot. At the same time, we used the “FindClusters” function to cluster the unsupervised units at 0.6 resolution on the same PC as for the “RunUMAP” function. Therefore, the datasets were visualized through UMAP plots. The closer the spot distance was, the closer the expression trend of spot was.

#### Differentially expressed gene analysis

The “FinAllMarkers” function is used to identify the differentially expressed genes (DEGs) in different clusters. Bonferroni correction method was used to adjust p value, and DEG with p value larger than 0.05 after correction was eliminated. The nonparametric Wilcoxon rank sum test in the “Seurat” software package was used to analyze the differential expression between subsets.

#### Cell cycle and differentiation analysis

Cells in each spot were divided into specific cell cycle and differentiation stage based on G2/M and S related gene expression (Table S2) and cell differentiation states (epithelium, p-EMT; Table S3). Briefly speaking, we used the “CellCycleScoring” and “AddModuleScore” function to calculate the cell cycle and differentiation score of each spot, and then matched it to the metadata.

#### Trajectory, pseudo-time, and immune analysis

The “Monocle2” software is used to identify differential genes that change between clusters or spots for developmental trajectory and pseudo-time analysis. The “Monocle2” package for trajectory and pseudo-time analysis contained 400 marker genes from the “differentialGeneTest” function that is designed to infer potential pedigree differentiation trajectories. A generalized additive model (GAM) is constructed to generate the average expression of each isotype. RNA counts of all spots in the cluster were selected as the input of “Monocle2” for downstream analysis. Additionally, we also used “AddModuleScore” function to evaluate the feature scores for immune infiltration enrichment based on the specific markers for T cells. B cells, NK cells and myeloid cells (Table S4).

## Results

### General profiles of spatial transcriptomics sequencing in hepatocellular carcinoma

**Figure 1:**
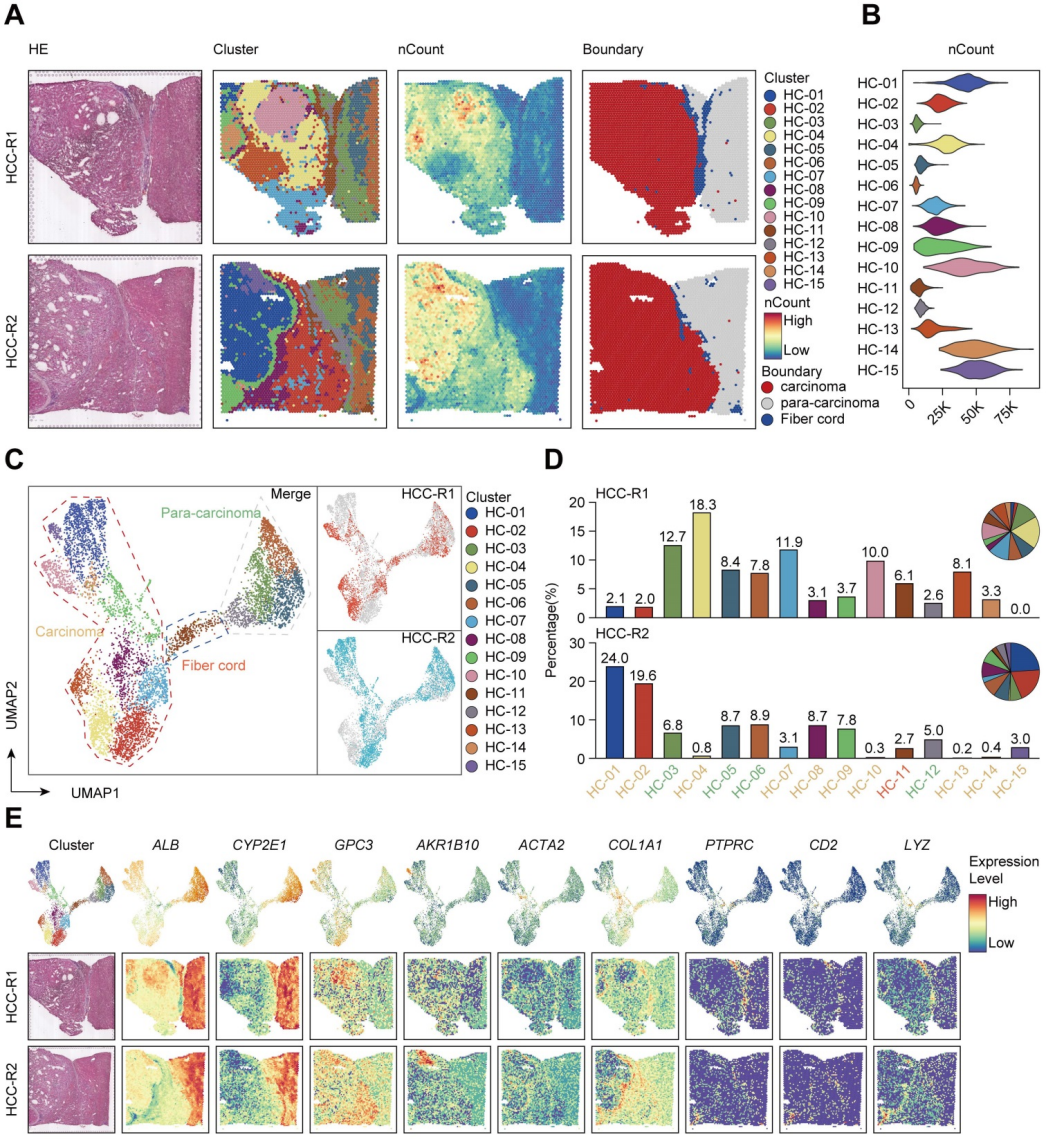
** Overall landscapes of spatial transcriptomics in hepatocellular carcinoma. A.** Spatial distribution of 15 clusters and nCounts as well as histological assessment and boundary among two regions of HCC (HCC-R1, R2). **B.** Violin plot demonstrating the number of UMI counts in 15 clusters. **C.** UMAP plot of all the spots from 15 clusters (colored by clusters and spatial locations; orange represents carcinoma, green represents para-carcinoma and red represents fiber cord). **D.** Bar plot showing the distribution of each cluster among two samples and pie plot indicating the proportion of clusters in each sample. **E.** Hematoxylin-eosin staining and spatial feature plots of marker genes in each sample.

To systematically explore the relations between spatial expression patterns and tumor heterogeneity in hepatocellular carcinoma, we randomly sampled tissues from two regions of surgically resected and pathologically confirmed HCC (HCC-R1, R2) of the same case with clearly demarcated and continuous cores, peripheral regions, and boundary regions. The fresh-frozen samples were then subjected to spatial transcriptomics sequencing via the 10× Genomics Visium platform with histological staining, cDNA sequencing library preparation, and further sequencing procedures. Hematoxylin-eosin staining revealed histological locations and boundaries; thus, we annotated them as sectors of carcinoma, para-carcinoma and fiber cord based on spatial locations (Figure 1A). We first integrated two samples and obtained 7553 specific captured areas (spots) on the ST arrays. Each spot reaches a length of 55 μm and contains a mixture of several cells that are not necessarily of the same cell type, which serves as an individual unit for further computational analysis and visualization. Data also showed that the mean number of nCount per spot was 22,387, and the mean gene count was 4,321 in this study, which qualified the sequencing data.

Next, we performed hierarchical clustering, and all the spots were further yielded into 15 clusters. We also investigated the spatial distributions of 15 clusters and nCounts among two samples. Generally, certain clusters (HC- 01, 02, 04, 07, 08, 09, 10, 12, 14, and 15) were in the carcinoma sector, while clusters (HC-03, 05, 06, and 12) were in the para-carcinoma sector and cluster HC-11 was in the fiber cord sector. It was also obvious that the number of nCounts in the carcinoma sector was larger than that in the para-carcinoma region and fiber cord sector, revealing a high abundance of gene expression in the tumor region (Figure 1A). All these clusters in the carcinoma sector indicated more nCounts than the clusters from other sectors (Figure 1B), which reflected a hyperproliferative state in the tumor region and was consistent with the results reported in previous studies 25. Uniform Manifold Approximation and Projection (UMAP) dimension reduction analysis depicted clear spatial segregation of spots belonging to specific clusters from the carcinoma, fiber cord and para-carcinoma sectors as the distance between the points represents the similarity between spots and spots of the same can form clustering, while spots of different clusters or subsets have obvious separation (Figure 1C), which further confirmed that various spatial regions cannot only be defined by histological assessments but also can be specifically distinguished from one another by spatial gene expression patterns. We displayed the distribution of 15 clusters among two samples and the results demonstrated that almost all the clusters existed in two samples. However, the proportion of the same cluster among two distinct samples are quite heterogeneous due to the discrepancy of spatial locations (Figure 1D).

To further characterize the identified clusters, we performed differentially expressed gene (DEG) analysis and profiled the featured DEGs in each cluster at the set of log-fold change (log FC) thresholds of 0.25 (Figure S1A). We examined the spatial expression of marker genes previously reported for common cell types in two samples to assess the sensitivity of the method of detecting the transcripts per spot, and the results confirmed that *ALB* and *CYP2E1* 14 were highly expressed in para-carcinoma regions; *GPC3* 26 and *AKR1B10* 27 in carcinoma regions; *ACTA2* and *COL1A1* (markers typically associated with activated fibroblasts or cancer-associated fibroblasts) 28 in the fiber cord and stromal regions, and the above also confirmed the reliability of ST sequencing results. We also detected the spatial distribution of *PTPRC* (leukocyte marker) 29, *CD2* (T cell and NK cell marker) 30, and *LYZ* (myeloid cell marker) 31 in two samples (Figure 1E); however, no evident spatial characteristics of these markers were found.

In the multi-step process of tumorigenesis, the basic biological functions of tumor cells have altered, such as the acquisition of unlimited replication and colonization ability and the activation of metastasis and invasion capacity 32. Consequently, we evaluated each spot or cluster for its likely cell cycle phases using signatures defined for G1, S, and G2/M phases based on functional annotations (Table S2). We found that most of clusters from the carcinoma sector had a higher proliferative capacity compared with those from the para-carcinoma and fiber cord sector (Figure S1B). We also appraised the differentiation origins of each cluster and undoubtedly found that the cluster HC-11 from fiber cord sector dominated the mesenchymal differentiation scores. Strikingly, clusters (HC-07, 08, and 09) originating from mesenchymal differentiation were situated in carcinoma sectors, revealing the epithelial-mesenchymal transition (EMT) process and potential malignancy of these carcinoma clusters (Figure S1B; Table S3).

Taken together, our recent analysis of spatial transcriptomics on two sections of HCC samples preliminarily reveals the discrepancies of gene expression patterns within the different regions of HCC microenvironment, providing new insights and novel strategies for exploring the relations between spatial gene expression and tumor heterogeneity of HCC.

### The spatial expression pattern of CCL15 in the tumor core region facilitates the HCC immunosuppressive microenvironment

**Figure 2:**
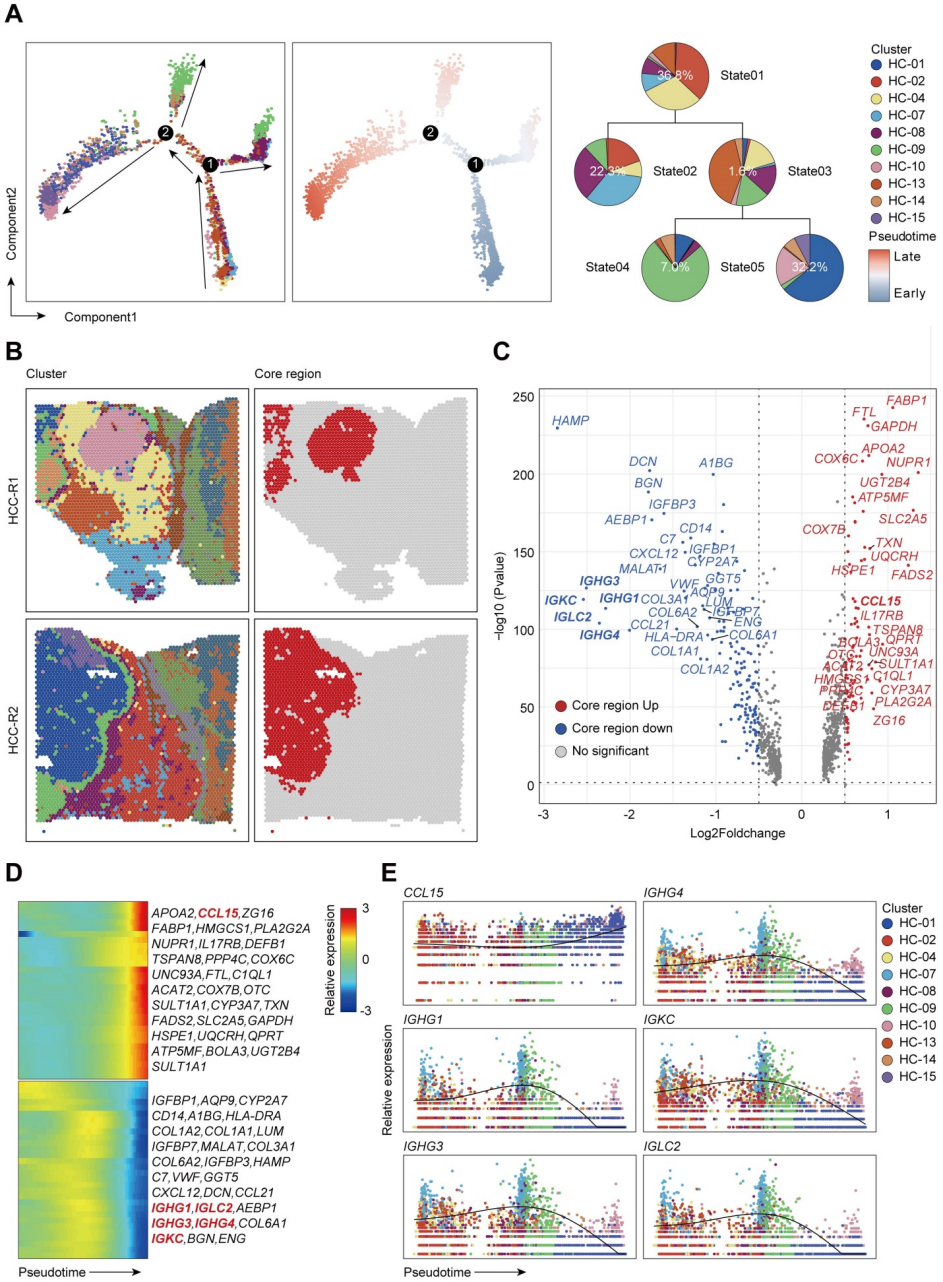
** The spatial expression pattern of CCL15 in the tumor core region facilitates the HCC immunosuppressive microenvironment. A.** Pseudo-time analysis and pie plots showing the developmental trajectory of spots from HCC-R1 & R2, colored by the clusters, states and pseudo-time. **B.** Spatial distributions of clusters in R1 & R2 as well as in the core region of HCC. **C.** Volcano plot of significantly differentially expressed genes in the core region of HCC. **D.** Heatmap displaying expression changes of differentially expressed genes in HCC-R1 & R2 along the pseudo-time trajectory. **E.** Scatter plots and fitting curves presenting the expression trend of selected marker genes in HCC-R1 & R2 along the pseudo-time trajectory.

Next, we intended to elaborate the spatial expression pattern and underlying mechanisms in distinct sectors. We set out to assess the differentiation and development trajectory of spots in the carcinoma sector using pseudo-time analysis and observed the developmental trajectory among clusters in HCC-R1/R2 (Figure 2A). Notably, clusters (HC- 01, 10, 14, and 15) were in the end stage of the developmental trajectory in HCC-R1/R2. All these clusters were also spatially located in the internal area of the carcinoma sector with the highest nCount, as shown before; hence, we annotated them as the core region of the carcinoma sector and displayed them among two samples (Figure 2B).

Subsequently, we speculated that inherent regularity of gene expression existed, especially in the core regions. We then analyzed and marked the top 20 DEG genes in tumor core regions and found that *IGHG1*, *IGHG3*, *IGHG4*, *IGKC*, and *IGLC2* (effector markers for humoral immunity) were notably downregulated in core regions, possibly indicating an immunosuppressive microenvironment (ISME). Among the upregulated genes presented, most of them (NUPR1, GSTA2, CCL15, UQCRH, GAPDH, and so on) have been documented previously to be elevated in HCC and play oncogenic roles in various aspects (Figure 2C) 33-36. Further analysis revealed that those downregulated genes were mainly expressed at the early stages in the trajectory of clusters from HCC-R1/R2 and diminished as the pseudo-time progressed, with the lowest expression at the end stage; however, nearly all the upregulated genes conversely increased gradually, and most of them were expressed in the end stages of the trajectory (Figure 2D). Interestingly, *IGHG1*, *IGHG3*, *IGHG4*, *IGKC*, and *IGLC2* were deficient at the end states of time, thus facilitating an immunosuppressive microenvironment (Figure 2E), while the reason why they were formed was unclear. Among those genes that dominated at the end of development trajectory, we noted that *CCL15*, an oncogenic chemokine, accumulated along the pseudo-time trajectory and was identified to facilitate the formation of tumor ISME (Figure 2E).

Given the limits of tissue samples, we further performed a secondary data analysis on a previous study of ST sequencing on primary liver cancers, which also presented spatial transcriptome map of three major liver cancer subtypes 23. In order to better match the spatial location of our tissue samples with higher spatial continuity that includes the carcinoma, fiber cord sector to para-carcinoma sector, we chose the leading-edge section of 4 HCC samples from their ST data and annotated them as HCC1, HCC2, HCC3, and HCC4 (Figure S2A). We reappraised histological boundaries, nCount profiles and performed clustering analysis on each tissue separately. We displayed spatial distributions of all the clusters among each tissue and HCC-1/2/3/4 can be clustered into 11, 9, 12, and 9 clusters, respectively. Notably, the number of nCounts in the carcinoma sector was remarkably larger than that in other sectors (Figure S2A). We also presented the spatial features and relatively quantified the levels of *CCL15* among 4 samples, which revealed that *CCL15* was obviously upregulated in the carcinoma sector of HCC1 and HCC4, however, no distinct and similar difference in HCC2 and HCC3 was observed (Figure S2B-C). Hence, we further analyzed the developmental trajectory of specific clusters pertaining to carcinoma sectors among HCC1 and HCC4 as previous mentioned (Figure S3A-B). *CCL15* was also increased as the pseudo-time progressed and was dominant in the end stages of the pseudo-time axis; Conversely, other key molecules such as *IGHG1*, *IGHG3*, *IGHG4*, *IGKC*, *IGLC2* decreased gradually among all the carcinoma sectors in HCC1 and HCC4 (Figure S3C-D). Taken together, their ST data on 4 pieces of HCC samples with spatial continuity supported our core conclusions and CCL15 did contribute to facilitating the HCC immunosuppressive microenvironment.

### CCL15 recruits and polarizes M2-like macrophages *in vitro* and *in vivo*

**Figure 3:**
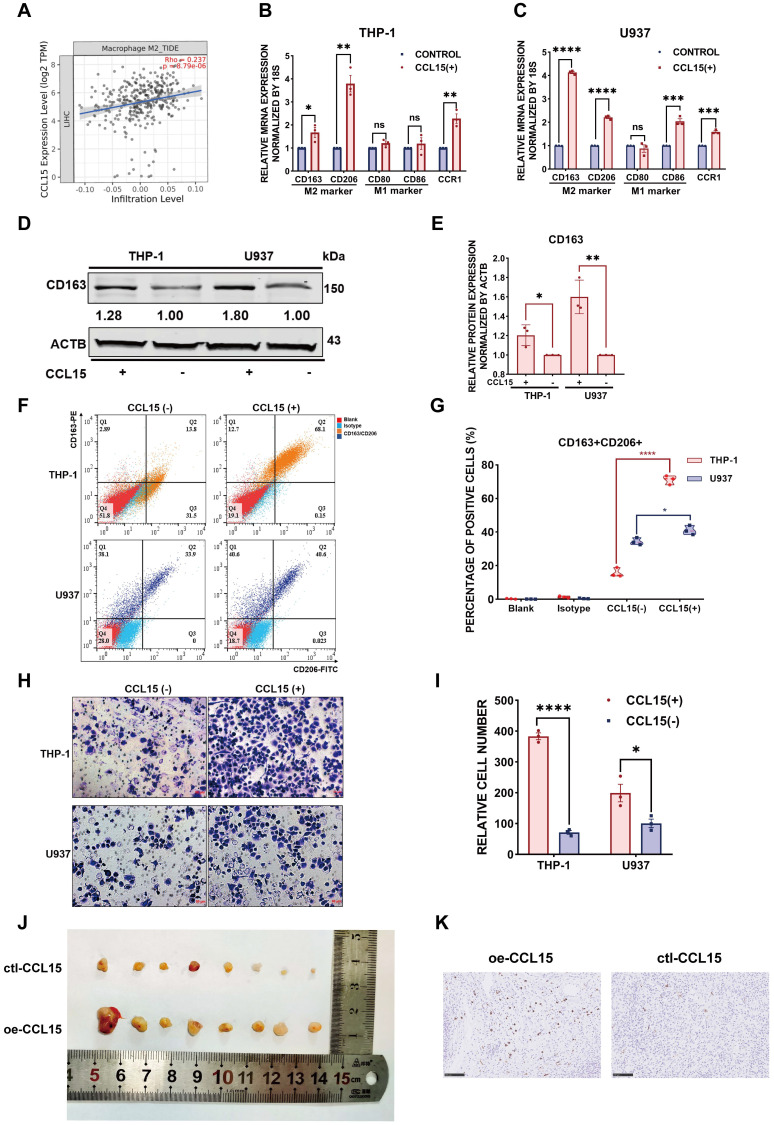
** CCL15 recruits and polarizes M2-like tumor-associated macrophages *in vitro* and *in vivo*. A.** Correlation between expression of CCL15 and infiltration of M2-like macrophages from the TIMER database. **B-C.** Relative mRNA levels of macrophage markers (M1, M2) and *CCR1* after treatment with CCL15 in THP-1 (**B**) or U937 (**C**) cells. **D-E.** Immunoblotting image (**D**) and relative quantitative analysis (**E**) of the M2-like macrophage marker CD163 after treatment with CCL15 in THP-1 and U937 cells. **F-G.** Flow cytometric image (**F**) and relative quantitative analysis (**G**) revealing the percentage of CD163^+^CD206^+^ M2-like macrophages after treatment with CCL15. **H-I.** Representative image (**H**) and quantitative result (**I**) of transwell migration assay of monocytes from THP-1 and U937 cells after treatment with CCL15 (scale bar, 50 µm). **J.** Representative image of subcutaneous xenografts resected from the oe-CCL15 group and control group. **K.** Immunohistochemical analyses of CD163 in the oe-CCL15 group and control group (scale bar, 100 µm).

Since the chemokine CCL15 was crucial for HCC immune microenvironment, we wondered how did CCL15 contribute to the formation of an immunosuppressive microenvironment that accordingly affected clinical outcome of HCC patients. To better investigate the potential mechanism of CCL15, we found that CCL15 was the most highly expressed in liver cancer among 21 solid tumors from the TCGA data (Figure S4A), indicating that CCL15 may play important roles in the progression of the liver tumor microenvironment (TME). Previous studies have shown that CCL15 can recruit CCR1^+^ bone marrow-derived inhibitory cells and CCR1^+^ neutrophils to promote liver metastasis of colorectal cancers 37-39. In another study, CCL15 can recruit suppressive CCR1^+^CD14^+^ monocytes into HCC tissues and promote immune escape by upregulating the expression of PD-L1, B7-H3, and IDO and activating STAT1/3, AKT, ERK, and other signaling pathways in an autocrine manner to promote HCC progression 35. We also analyzed their comparison data and found that CCR1^+^CD14^+^ monocytes recruited by CCL15 showed significantly higher expression levels of the M2-like macrophage markers *CD163L1* and *CD200R* 35, suggesting the potential of CCR1^+^ monocytes recruited by CCL15 to polarize toward the M2-like type. Importantly, we observed a positive correlation between CCL15 and the infiltration degree of M2-like macrophages in the TIMER database (Figure 3A). M2-like macrophages secrete various growth factors, cytokines, and collagenases and consequently promote tumorigenesis and tumor development 40. Taken all these standpoints into account, we hypothesized that CCL15 may be associated with M2-type macrophages and synergistically facilitate the immunosuppressive microenvironment of HCC. To verify the hypothesis, we next constructed macrophage models *in vitro* with THP-1 or U937 cell lines stimulated by phorbol ester (PMA), which increases the expression of macrophage markers (Figure S4B). After pre-experimentation to determine the optimal stimulating concentration of CCL15 (Figure S4C-D), we found that the markers of M2-like macrophages and their receptor CCR1 were upregulated at both the transcript and protein levels (Figure 3B-E). Flow cytometric analysis showed that the proportion of CD163^+^CD206^+^ positive cells increased compared to the control group after CCL15 stimulation, revealing a trend of macrophage polarization toward the M2-like type (Figure 3F-G). Furthermore, transwell migration assays showed an enhancement in the migration ability of macrophages in the presence of CCL15, indicating that CCL15 enhances the chemotaxis capacity of macrophages (Figure 3H-I). To verify the results *in vivo*, we also propagated massive Huh7 cells overexpressing CCL15 stably and applied subcutaneous tumorigenesis model with nude mice. We found that overexpressing CCL15 can increase the vitality and tumor growth of xenograft tumors (Figure 3J, Figure S4E-F). Intriguingly, the expression of CD163 in the overexpressing group was higher than that in the control group, revealing a higher infiltration of CD163 macrophages in the xenograft tumors after overexpressing CCL15 (Figure 3K). Collectively, these observations demonstrated that CCL15 can recruit monocytes and polarize them toward M2-like macrophages *in vitro* and *in vivo*, but further mechanism study is still demanding.

### Combined predictive role of CCL15 and CD163 in the worse prognosis of HCC patients

**Figure 4:**
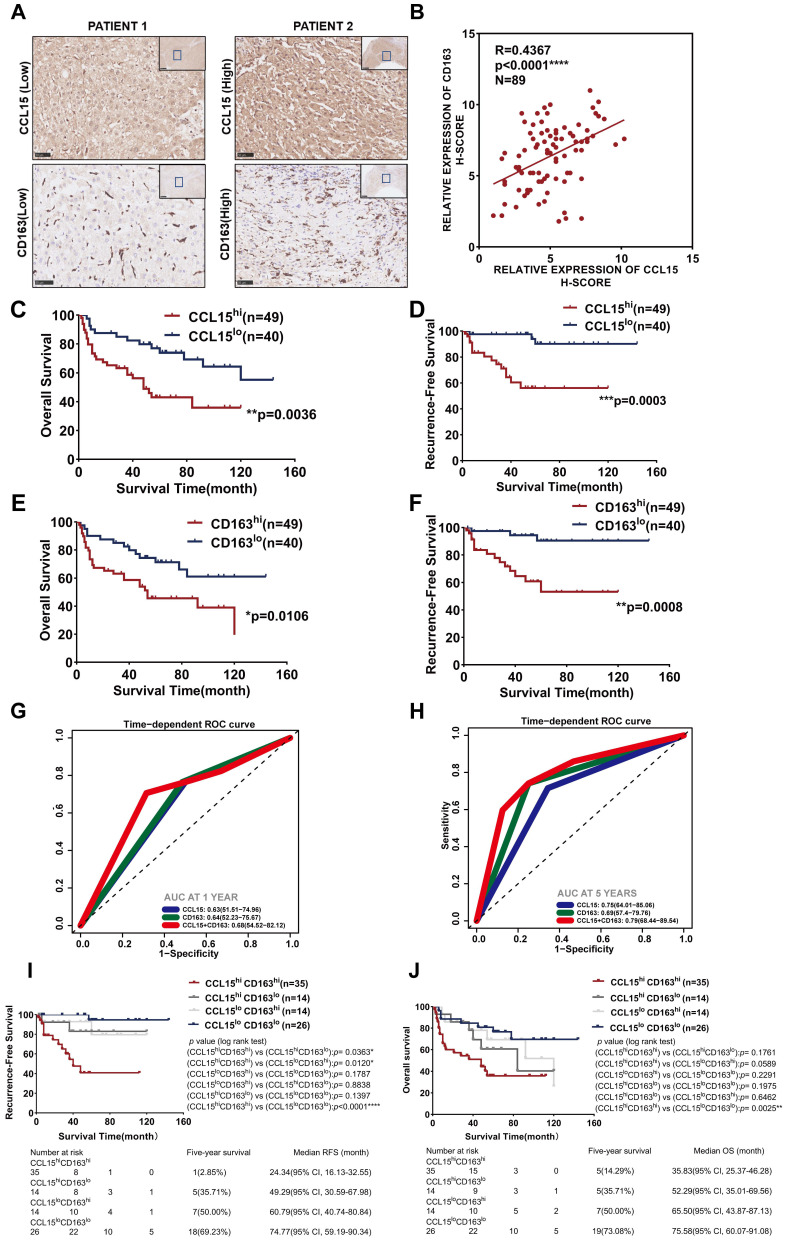
** Combined predictive roles of highly-expressed CCL15 and CD163 predict the worst prognosis of HCC patients. A.** Representative immunochemical staining of CCL15 and the M2-like macrophage marker CD163 in 89 patients (scale bar, 50 µm). **B.** Correlation between the relative expression of CCL15 and CD163 using the H-score method. **C-D.** Kaplan-Meier survival curves showing correlation between CCL15 alone and OS (**C**) and RFS (**D**). **E-F.** Kaplan-Meier survival curves showing correlation between CD163 alone and OS (**E**) and RFS (**F**). **G-H.** ROC analyses showing combined predictive value of CCL15 and CD163 for OS at 1 year (**G**) and 5 years (**H**). **I-J.** Kaplan-Meier survival curves showing the worst OS (**I**) and the worst RFS (**J**) in the CCL15^high^CD163^high^ group.

Additionally, we further detected the expression of CCL15 and the M2-like macrophage marker CD163 in HCC tissues of 89 patients from the CH cohort (Figure 4A). The results indicated a positive correlation (R=0.4367, p<0.0001) between CCL15 and CD163 expression levels (Figure 4B). After determining the optimal cutoff values using X-tile software (Version 3.6.1), both CCL15 and CD163 correlated significantly with prognosis, and the higher the levels of CCL15 or CD163 were, the worse the OS and RFS of the patients (Figure 4C-F). Furthermore, the combined predictive value of CCL15 and CD163 was higher, with an area under the curve (AUC) of 0.68/0.79 for one/five years of OS, than that of CCL15 alone (AUC area of 0.63/0.75) and CD163 alone (AUC area of 0.64/0.69) (Figure 4G-H), which indicated the superior value of them in predicting the prognosis. Therefore, we further evaluated the relationship between combined application of the CCL15 and CD163 expression and the prognosis of HCC patients. Based on the expression differences, all the patients can be classified into CCL15^hi^CD163^hi^ group, CCL15^hi^CD163^lo^ group, CCL15^lo^CD163^hi^ group, CCL15^lo^CD163^lo^ group, respectively. We next compared the relationship of survival prognosis among these four groups, and found the most significant survival difference between the CCL15^lo^CD163^lo^ group and the CCL15^hi^CD163^hi^ group. CCL15^hi^CD163^hi^ group was blessed with the worst prognosis, verifying the predictive superiority of joint application of CCL15 and CD163. The five-year survival rate (14.29%) and median survival time (35.83, 95% CI: 25.37-46.28) for OS and the five-year survival rate (2.85%) and median survival time (24.34, 95% CI: 16.13-32.55) for RFS of the CCL15^hi^CD163^hi^ group were the worst among the four groups (Figure 4I-J). However, we did not observe obvious significance in the clinical characteristics between the CCL15^hi^CD163^hi^ and CCL15^lo^CD163^lo^ groups due to the limits of sample capacity (Table S5).

Taking the above results into account, our results suggest that CCL15 may promote the formation of immunosuppressive microenvironment and affect the prognosis of HCC patients by recruiting and polarizing M2-like macrophages. Furthermore, the high expression of CCL15 and M2-type macrophage marker CD163 predicts poor survival prognosis, and the combined application of CCL15 and CD163 expression has better prognostic value. These results further enrich the function and role of CCL15 in the immune microenvironment of HCC, but the mechanisms driving tumor progression by CCL15 and M2-likes macrophages still need further discussion.

### CCL19 and CCL21 share similar expression patterns and are remarkable in the immune infiltration enrichment (IIE)

**Figure 5:**
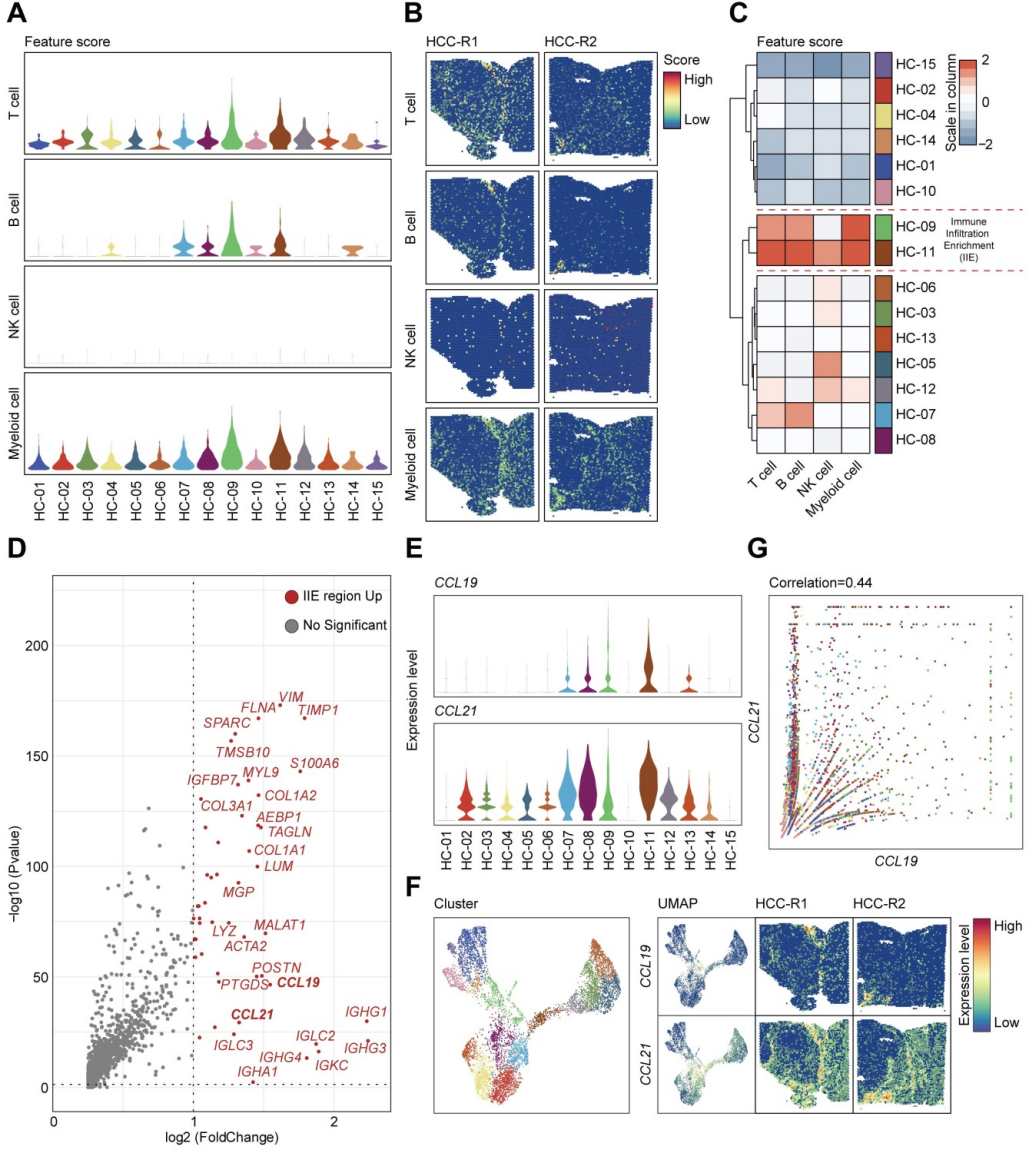
** CCL19 and CCL21 sharing similar expression patterns and remarkable in immune infiltration enrichment (IIE) contribute to the abundance of immune cells in HCC. A.** Violin plots showing the feature scores of immune cells such as T cells, B cells, NK cells and myeloid cells among 15 clusters. **B.** Spatial distribution of T cells, B cells, NK cells and myeloid cells in two samples. **C.** Heatmap indicating the overall infiltration of immune cells in each cluster. **D.** Volcano plot of significantly upregulated genes in the IIE. **E.** Violin plots showing the expression levels of CCL19 and CCL21 among 15 clusters. **F.** Spatial features of CCL19 and CCL21 in two samples. **G.** Correlation between the relative expression of CCL19 and CCL21 among 15 clusters.

Given that cellular components of tumor microenvironment are fairly complex, with distinct populations of immune cells playing vital roles in the progression of HCC as well as immunotherapy, recent studies have tried to elaborate the characteristics and functions of T cells, tumor-associated macrophages (TAMs), and dendritic cells (DCs) in HCC 11, 12, but the global landscapes of immune cells are still poorly understood. In our studies, we first scored the abundance of immune cells such as T cells, B cells, natural killer cells, and myeloid cells annotated by cell marker signatures (Table S4) among 15 clusters (Figure 5A) and characterized the spatial expression patterns of immune cells among two samples (Figure 5B). T cells and B cells seemed to be remarkably infiltrating in specific areas or clusters among the tissues; NK cells presented a low abundance in HCC and spatially localized in a random regularity; Myeloid cells were the most abundant immune cells but without apparent discrepancy in spatial distributions due to the limits of sequencing length, and consequently we cannot distinguish specific myeloid subsets such as monocytes, macrophages and other cell types (Figure 5B). After hierarchically clustering the abundance of immune cells, clusters (HC-09, and 11) were notably enriched with immune cells compared with other clusters and were well integrated together, which indicated the most enrichment of immune cells such as T cells, B cells and myeloid cells (Figure 5C); thus, we defined the aggregation of these clusters as immune infiltration enrichment (IIE).

We investigated the significantly upregulated genes in the IIE and observed that *IGHG1*, *IGHG3*, *IGHG4*,* IGLC2*, *IGKC*,* IGHA1*,* IGHM* and other molecules were dramatically elevated (Figure 5D), which in turn confirmed the aggregation of B cells in these clusters (Figure 5A-C). However, we still wondered what triggered the accumulation of immune cells in the IIE. We noticed that *CCL19* and *CCL21* were also obviously prominent among the upregulated genes. Both CCL19 and CCL21 are derived from a population of identical cells, such as various stromal cells within primary and secondary lymphoid organs, T lymphocytes, and lymphatic endothelial cells, in peripheral tissues 41. CCL19 and CCL21 are mainly involved in the homing, migration process and maturation of dendritic cells, as well as in the activation, recruitment and recirculation of T lymphocytes and B cells in the adaptive immune system 42, 43. Moreover, several studies have indicated that CCR7-CCL19/CCL21 has an antitumor function by recruiting T lymphocytes and dendritic cells in lung cancer and other malignant tumors 44, 45, and few have been reported in HCC yet. We therefore hypothesized that upregulated CCL19 and CCL21 may be the leading cause of the enrichment of immune cells in the IIE. We further detected the expression levels and spatial distribution of *CCL19* and *CCL21* among 15 clusters and two pieces of samples (Figure 5E-F). In accordance with previous results, *CCL19* and *CCL21* shared similar spatial expression patterns, and both were mainly in the fiber cord sector (Figure 5F). Although the expression of *CCL21* was much higher than that of *CCL19*, *CCL19* was positively correlated with *CCL21* among 15 clusters in two samples (Figure 5G).

**Figure 6:**
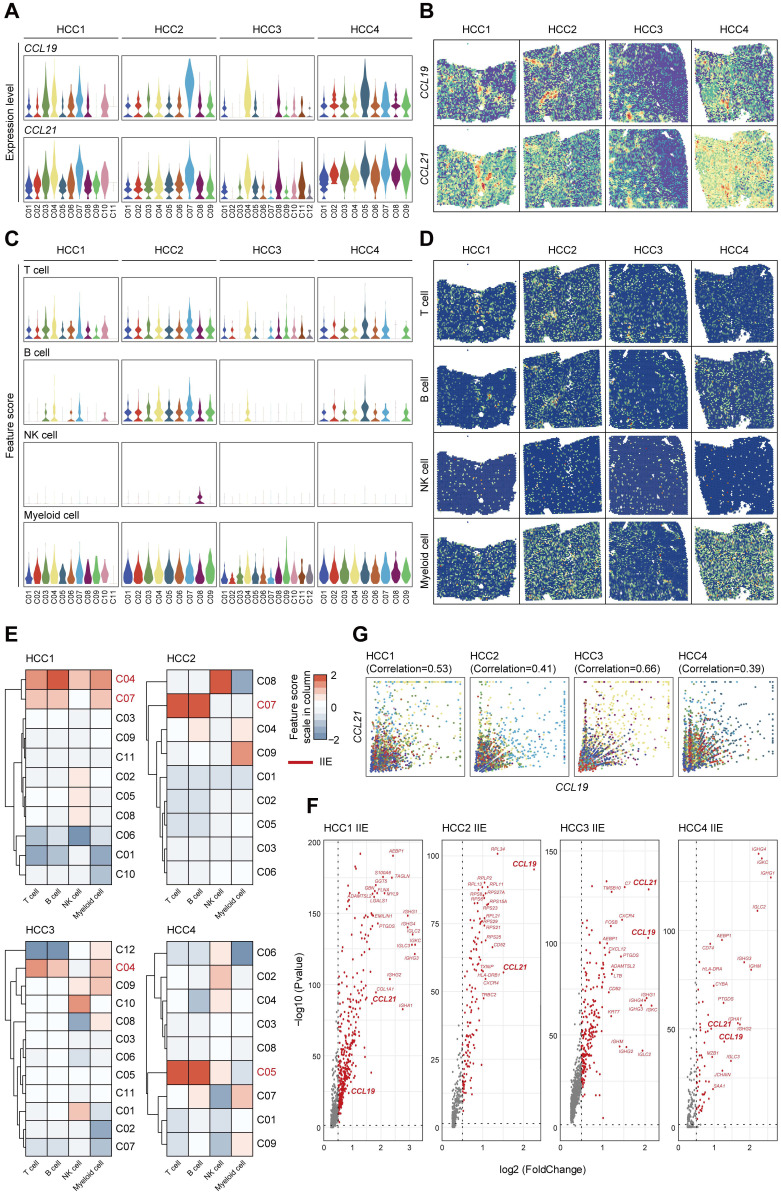
** Highly-correlated CCL19 and CCL21 are indeed dominant in the IIE from secondary data analysis of previous ST sequencing on HCC. A.** Violin plots showing the expression levels of *CCL19* and *CCL21* among all the clusters from four various samples. **B.** Spatial distribution of *CCL19* and *CCL21* among four samples. **C.** Violin plots showing the feature scores of immune cells such as T cells, B cells, NK cells and myeloid cells among all the clusters from four various samples. **D.** Spatial distribution of T cells, B cells, NK cells and myeloid cells among four samples. **E.** Heatmap indicating the overall infiltration of immune cells in each cluster and the IIE in each sample (the clusters marked red).** F.** Volcano plot of significantly upregulated genes in the IIE among four samples. **G.** Correlation between the relative expression of CCL19 and CCL21 in all the clusters among each sample.

To comprehensively demonstrate the functions of CCL19 and CCL21 among immune microenvironment, we further investigated the expression levels and spatial distributions of *CCL19* and *CCL21* among the clusters from the leading edging section of 4 pieces of HCC samples from our secondary data analysis (Figure 6A-B) on previous ST data 23. We also detected the infiltration degrees and spatial distributions of immune cells and observed that T cells and B cells were mainly enriched in the fiber cord sector (Figure 6C-D), which is in accordance with the spatial expression patterns of *CCL19* and *CCL21*. After hierarchically clustering the abundance of immune cells, we further defined the IIE among 4 samples (Figure 6E). Consistent with our ST data, *CCL19* and *CCL21* were dominant in the up-regulated genes among the IIE (Figure 6F). Furthermore, the level of *CCL19* was positively correlated with CCL21 among the clusters from 4 samples respectively (Figure 6G). Conclusively, CCL19 and CCL21 may also function in synergistic effects on biological processes among the IIE region in the spatial architecture of HCC due to their similarity in spatial expression patterns and interactive approaches, which was reliably confirmed by previous ST data as well.

### CCL19 and CCL21 inhibit the growth of HCC by enriching the abundance of T cells and B cells

**Figure 7:**
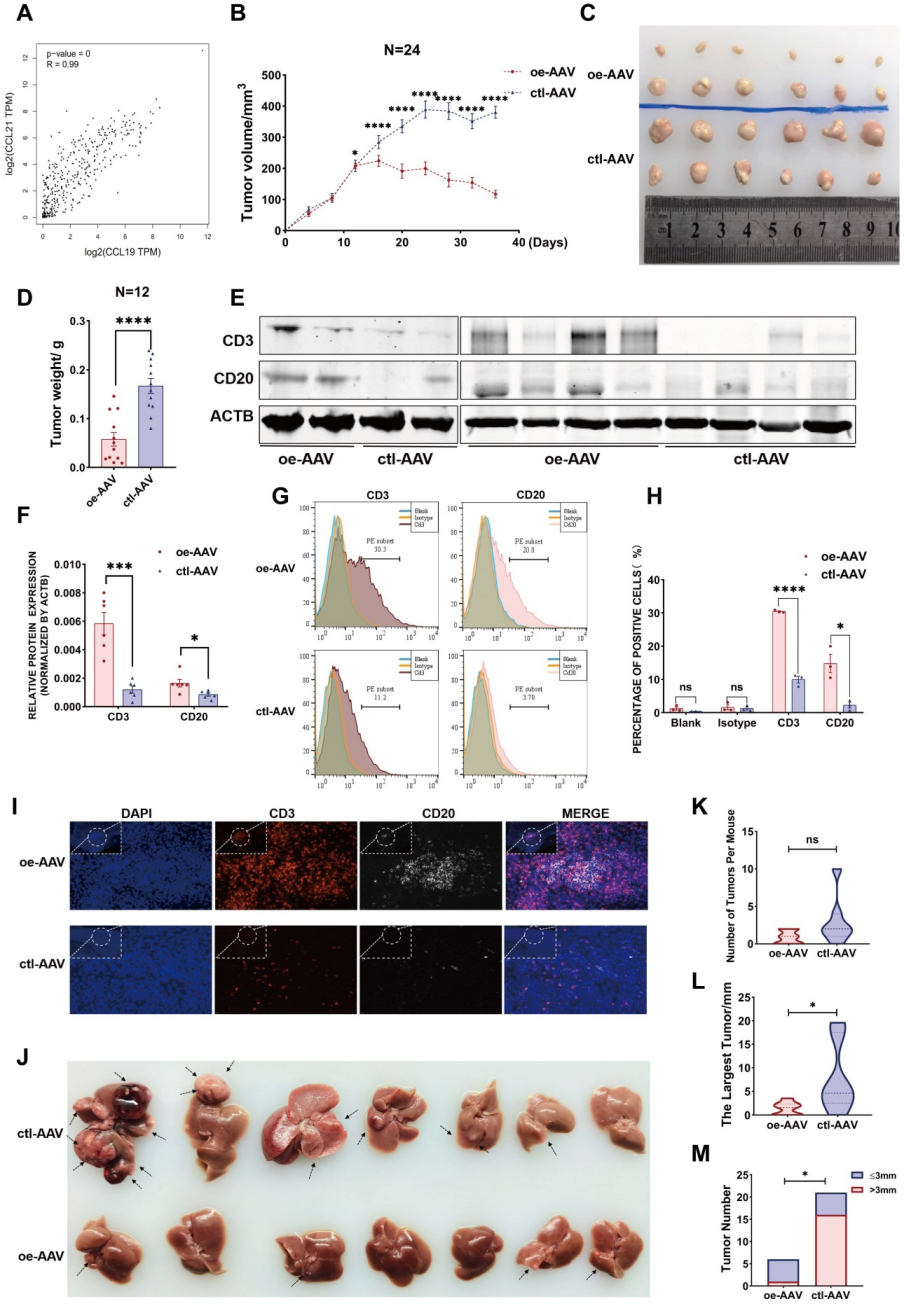
** CCL19 and CCL21 inhibit the growth of HCC by enriching the abundance of T cells and B cells. A.** Correlation between expression of CCL19 and CCL21 in HCC from TCGA database (R=0.99. p-value=0). **B.** Tumor volume changes of subcutaneous xenografts after hepa1-6 cell clones were implanted, n=24. **C.** Representative image of subcutaneous xenografts resected from the oe-AAV group and control group (n=12). **D.** Tumor weight of subcutaneous xenografts from the two groups. **E-F.** Immunoblotting image (**E**) and relative expression (**F**) showing CD3 and CD20 in subcutaneous xenografts of the two groups (n=6). **G-H.** Flow cytometric image (**G**) and relative quantitative analysis (**H**) indicating CD3^+^ and CD20^+^ cells in the two groups. **I.** Immunofluorescence image showing the infiltration of CD3^+^ T cells and CD20^+^ B cells in the two groups (scale bars 20 µm; 10 µm). **J.** Resected liver image showing tumor growth of DEN/CCl_4_-induced HCC after tail vein injection of AAV (n=7). **K.** Comparison of total tumor number between the oe-AAV group and the control AAV group from DEN-CCl_4_-induced HCC models. **L.** Comparison of the largest tumor diameters between the two groups. **M.** Comparison of the number of tumor diameters (>/≤3 mm) between the two groups.

Considering their similarities in spatial expression patterns in our ST data, we have strikingly verified their positive correlation from our second data analysis (Figure 6G), which was also strongly confirmed with a coefficient of 0.99 from the TCGA database (Figure 7A), reflecting a potential synergistic effect between CCL19 and CCL21. Moreover, we analyzed the predictive roles in prognosis with combined CCL19 and CCL21 in HCC and observed that high expression of CCL19 and CCL21 predicts a good prognosis in liver cancer from the TCGA database (Figure S5A), suggesting that combined CCL19 and CCL21 may be used as a new strategy for HCC immunotherapy. To evaluate the potential therapeutic effects and possible mechanisms, we next constructed vectors of adeno-associated viruses (AAV-8) overexpressing *Ccl19*, *Ccl21a* and combined *Ccl19*/*Ccl21a*, which target hepatic cells specifically, and evaluated therapeutic effects in the subcutaneous xenograft models respectively. Results showed that overexpressing combined *Ccl19*/*Ccl21a* can remarkably retard tumor growth and decrease tumor vitality of hepa1-6 cells, while overexpressing *Ccl19* or *Ccl21a* alone showed a slightly significant difference of tumor growth compared to the control group (Figure S5B-D), indicating that overexpressing combined *Ccl19* and *Ccl21a* elicited a synergistic effect in inhibiting the tumor growth. Hence, we next focused on the combined therapeutic effects of *Ccl19*/*Ccl21a* with further research. Besides subcutaneous tumor models, we also built DEN/CCl_4_-induced liver cancer models, and the timeline and procedures are shown (Figure S5E). Specifically, we chose C57/BL6 mice rather than SCID or nude mice for the experiments due to the deficiency in normal immune functions of the latter types.

In the subcutaneous xenograft model, hepa1-6 cells were first injected into the bilateral armpits of 24 mice, and the growth of emerging tumors was dynamically monitored every four days. Ccl19/Ccl21a-overexpressing AAV (oe-AAV) and control AAV were then intratumorally injected when the largest diameter of the xenograft reached 5 mm. The results showed that the growth of tumors from the oe-AAV group was slowed and the tumor volume was gradually reduced, whereas the tumor growth and tumor volume of the control AAV group continued to increase (Figure 7B). The weight and volume of tumors in the oe-AAV group were smaller than those in the control group (Figure 7B-D), indicating that CCL19 and CCL21 have a therapeutic effect in inhibiting the growth of HCC. Previous studies showed that CCL21 can affect tumor progression by recruiting immune cells such as T lymphocytes 46, 47, and have reported that tumor-infiltrating B cells may inhibit liver cancer progression and improve prognosis by interacting with CD4^+^ T cells in close proximity and subsequently activating CD8^+^ T cells, but the origins of tumor-infiltrating T cells and B cells are still unclear. Therefore, we hypothesized that CCL19 and CCL21 influenced the infiltration degree of T cells and B cells via recruitment.

We first evaluated relations between the levels of CCL19 and CCL21 and infiltration of immune cells in the TIMER database and found that CCL19/CCL21 correlated positively with CD4^+^ T cells, CD8^+^ T cells and B lymphocytes (Figure S5F), particularly with naïve T cells, central memory T cells, effector T cells and Th-1-like cells (Figure S5G, other data not shown). We further validated the correlation of *Ccl19* with *Cd3e*, *Cd4*, *Cd8a*, *Cd19*, *Cd20*, and *Ifng* (another marker of T cell activation) in subcutaneous xenografts, and the results showed that *Ccl19* correlated positively with *Cd3e*, *Cd19*, *Cd20*, and *Ifng* (Figure S5H). Furthermore, the T cell marker CD3 and the B cell marker CD20 were upregulated at the protein level in the oe-AAV group compared to the control group (Figure 7E-F). Flow cytometric analyses of tumor xenografts revealed a higher proportion of CD3^+^ cells and CD20^+^ cells in the oe-AAV group than in the control group (Figure 7G-H), and tissue immunofluorescence results confirmed significantly higher infiltration of CD3^+^ T cells and CD20^+^ B cells in the oe-AAV group (Figure 7I).

In another model, mice were tail vein injected with oe-AAV and control AAV after DEN/CCl_4_-induced liver tumors arose, and the mice were sacrificed after three months (Figure 7J). The results indicate no significant difference in the overall tumor number (Figure 7K), while the maximum tumor volume and the number of tumors larger than 3 mm were conspicuously reduced in the oe-AAV group compared with the control AAV group (Figure 7L-7M). Taken together, these observations indicate that CCL19 and CCL21 inhibit the growth of HCC by enhancing the infiltration of tumor-infiltrating T cells and B cells. Thus, overexpression of *Ccl19*/*Ccl21a* AAV may be used as a novel strategy for immunotherapy of HCC, but further analysis is essential.

## Discussion

HCC is a highly heterogeneous and malignant tumor, and the relationship between HCC heterogeneity, microenvironment and spatial location has been widely discussed 23, 48, 49. Most of the previous studies have focused on the overall gene expression of mixed cells in carcinoma and para-carcinoma areas, but the accuracy of gene expression is insufficient enough for detailed and in-depth studies 22, 50-52. As sequencing techniques advances, single cell transcriptomics can analyze the function of cell subsets at the level of single-cell resolution 14, 53, but spatial specificity of gene expression cannot be determined due to the absence of spatial information of intricate tissue structures, while spatial transcriptomics sequencing can quantify and localize gene expression, decipher the innate correlations of spatially correlated genes and accordingly tackle with this problem properly 54.

In our study, we conducted ST sequencing on two pieces of HCC specimens from one patient. Each piece of tissues comprised distinct but continuous regions from carcinoma tissue and the tumor boundary region to para-carcinoma tissue. We first described overall landscapes of spatial gene expression patterns among two samples and intratumor heterogeneity. Next, we defined the tumor core region and the IIE via bioinformatics analysis, which was also confirmed by our secondary data analysis on previous study focusing on tumor heterogeneity of primary liver cancers 23. Finally, we further validated the analysis results through the molecular, cellular, animal experiment and clinical tissue samples, the basic conclusions are as follows: (1) All spots were categorized into 15 subgroups by hierarchical clustering, differentially expressed genes and dimension reduction analysis, and their relationship with spatial location was determined; (2) Malignant subsets with intratumor heterogeneity phenotypes were identified by nCount, cell cycle phases, and epithelial-mesenchymal feature analysis; (3) Specific immune-related spatial regions, such as the tumor core region and the IIE, were defined by the pseudo-temporal analysis of tumor subsets and spatial expression patterns of immune cells; (4) CCL15 was significantly upregulated in the tumor core region, and promoted the formation of immunosuppressive microenvironment by recruiting and polarizing M2-like macrophages *in vitro* and *in vivo*; (5) High expression of CCL15 and CD163 respectively predicts poor prognosis of HCC patients, and the combined application has better predictive value; (6) Highly-correlated CCL19 and CCL21 were synergistically upregulated in the IIE and inhibited the growth of HCC by increasing the infiltration degree of T cells and B cells.

Concretely speaking, we profiled the overall landscapes of hepatocellular carcinoma with ST sequencing and all the 7553 spots were classified into 15 clusters. We then determined the features of 15 clusters belonging to distinct sectors by virtue of the histological boundaries, marker genes and differential gene expression, cell cycle phases and differentiation origins. Intratumor heterogeneity, especially immune-ITH, has been documented to impact the clinical outcomes of HCC, which is also characterized by an increased immunosuppressive or exhaustive TME; However, spatial distribution characteristics and expression of other cell markers in the tissue structure of HCC still need further consideration, and the origins and contributing factors for ITH are still largely unknown 55. Accordingly, we next sought to explore relations between ITH and spatial expression patterns using our ST data and found that those clusters located in the core area of the carcinoma sector are also in the end stages of the pseudo-time trajectory. Consequently, we defined these clusters as tumor core region where most of the elevated DEGs were reported previously to play oncogenic roles in HCC and those significantly downregulated genes feature with humoral immunity enhancement, which jointly facilitate the formation of immunosuppressive microenvironment. Among the upregulated genes, we noted that CCL15 may be the culprit for this condition. The above results were also verified by leading-edge tissues of four HCC section samples from our secondary data analysis on a previous study 23. Further studies revealed that CCL15 can recruit and polarize M2-like macrophages *in vitro* and *in vivo*. Clinical relevance analysis also indicated that high expression of CCL15 or the M2-like macrophage marker CD163 predict a poor prognosis of HCC and the combined predictive value of CCL15 and CD163 in the prognosis is superior to that using either marker alone. Currently, macrophage polarization involves a variety of molecular mechanisms, including TLR4/NF-κB, JAK/STATS, TGF-β/SMADS, PPARγ, NOTCH and microRNA signaling pathways 56, however, the detailed mechanism driving M2-like macrophages by CCL15 will be our future work.

Furthermore, we also evaluated the infiltration scores and spatial distributions of various immune cells, including T cells, B cells, NK cells and myeloid cells among 15 clusters. Clustering results showed that two clusters are rich in immune cells especially T cells and B cells, and are annotated as immune infiltration enrichment. Contrary to the conditions of tumor core region, upregulated DEGs in the IIE are characterized by immunoglobin families and two prominent chemokines, CCL19 and CCL21, and the results are verified by our second data analysis on a previous study 23.

Further studies elucidated their strong correlations and similarities in spatial expression pattern, biological process and clinical significance of HCC. *In vivo* experiments of AAV showed that high expression of CCL19 and CCL21 inhibits the growth of HCC by influencing the infiltration of CD3^+^ T cells and CD20+ B cells in the subcutaneous xenograft model, while there was a slight significance in the DEN/CCl4-induced tumor models when compared with tumor sizes in two groups. The reason may well be that CCL19 and CCL21 function better in the tumor progression rather than the tumorigenesis process of HCC, and DEN/CCl4-induced tumor models are not strongly consistent due to individual differences among mice and the sample sizes. Anyway, the above conclusions indicate good prognosis and may serve as a novel therapeutic target for HCC immunotherapy. Taken together, we determined the specific chemokines in either the tumor core region or IIE, which have remarkably different roles in clinical applications due to spatial heterogeneity in gene expression. Notably, the recruiting mechanism of these chemokines to immune cells still needs further exploration.

In conclusion, our study comprehensively reveals intratumor heterogeneity, especially immune-ITH, in HCC based on spatial transcriptomics technology. We further reveal the spatial expression patterns in specific regions of some key molecules such as CCL15, CCL19, and CCL21, which affect the infiltration and recruitment of various immune cells and collectively promote intratumor heterogeneity in the HCC microenvironment, thus influencing the prognosis of HCC patients. Our studies also highlight the clinical significance of the spatial heterogeneity and gene expression patterns, laying the foundation for developing new prognostic markers and therapeutic strategies for HCC.

## Supplementary Material

Supplementary methods, figures and tables.

## Abbreviations

AAV: adeno-associated virus
AUC: area under curves
DCs: dendritic cells
DEGs: differentially expressed genes
EMT: epithelial-mesenchymal transition
FFPE: formalin-fixed paraffin-embedded
HCC: hepatocellular carcinoma
HE: hematoxylin-eosin staining
IIE: immune infiltration enrichment
ISME: immunosuppressive microenvironment
ITH: intratumor heterogeneity
OCT: optimal cutting temperature
OS: overall survival
PLC: primary liver cancer
RFS: recurrence-free survival
ROC: receiver operating characteristic curve
sc-RNA seq: single cell RNA sequencing
ST: spatial transcriptomics
TAMs: tumor-associated macrophages
TME: tumor microenvironment
UMAP: uniform manifold approximation and projection
UMI: unique molecular identifier
