# Supplementary material for: Spatial maps of hepatocellular carcinoma transcriptomes reveal spatial expression patterns in tumor immune microenvironment
Source: Theranostics. 2022 May 16;12(9):4163–80. doi: 10.7150/thno.71873 (PMC9169356; doi:10.7150/thno.71873)
Supplement: Supplementary file 1 — Supplementary methods, figures and tables. [file thnov12p4163s1.pdf]

Supplementary Materials and Methods

Cell lines

Huh7, THP-1, and U937 Cells were cultured in DMEM or RPMI 1640 medium (Gibco, USA) containing 10% FBS (Gibco, USA), 1% penicillin, streptomycin (Invitrogen, USA) and anti-prophylactic treatment (Invivogen, USA) with a 5% CO2 incubator at 37 ℃. Extra 1‰ β-mercaptoethanol was added into culturing medium for THP-1 or U937 cells. Relative cellular assays were shown as follows.

Mice

Nude mice or C57/BL6 mice used in this study were all male, six-eight weeks old. All mice were kept under specific pathogen-free conditions at room temperature with a 12 h light/dark cycle, with food and water were provided ad libitum. Animal studies were approved by the Institutional Animal Care and Use Committee of Naval Medical University (Shanghai, China).

Total RNA isolation, reverse transcription PCR and quantitative real-time PCR

All the reagents and consumables are pretreated with Rnase removal. Total RNA was extracted from cells and tissues by TRIzol reagents (Thermo Fisher, USA) or RNA isolation kit (Vazyme#R701, Dalian, China). The concentration and quality of RNA was assessed by Nandrop 2000 (Thermo Fisher). RNA was stored at -80 ℃ until use and complementary DNA was synthesized with reverse transcription reagents (Takara#RR036A) according to the

manufacturer's instructions. Quantitative real-time PCR (qRT-PCR) was used to detect the relative transcription expression of target genes using ChamQ Universal SYBR qPCR Master Mix (Vazyme#Q771-02). The samples were normalized to the expression of 18S or β-action using $2^{-\Delta\Delta CT}$ method. The sequence of primers was shown in Table S6.

Protein extraction and western blotting (WB)

Briefly, proteins were extracted from cultured cells and fresh frozen tissues with RIPA lysis buffer (Beyotime, China) according to the manufacturer's instructions. All the procedures were operated on ice. Then the lysates were separated by 10% SDS-polyacrylamide gel electrophoresis and transferred to nitrocellulose membranes. The membranes were immersed in quick block buffer (Epizyme, China) and then probed overnight at 4°C in solutions with primary antibodies, and the details of antibodies were shown on Table S7. Next, the membranes were incubated with fluorescent secondary antibodies after the wash-up of primary antibodies. Finally, protein levels were detected and evaluated by Odyssey infrared fluorescent scanner (LICOR, America). The relative expression levels were normalized to the endogenous control (β-actin) and quantified on grey values.

Immunohistochemistry and immunofluorescence

Human HCC tissues and subcutaneous tumor tissues were fixed in formaldehyde and embedded in paraffin for further studies. In immunohistochemistry (IHC) assays, tissue sections were dewaxed, subject to

antigen retrieval, blocked with 10% goat serum and then incubated with primary antibodies overnight at 4 ℃ as well as secondary antibodies at 37 ℃ for 45 mins, the details of antibodies were listed at supplementary Table S7. DAB treatment, hematoxylin staining and hydrochloric acid alcohol differentiation was used to dye nuclei. Finally, the sections were scanned by white light scanner or observed under the microscope. The relative quantification of targets in IHC depended on H-score, which integrated the percentage of positive cells and staining strength of targeted proteins.

As for immunofluorescence (IF) assays with two targets, the procedures before incubating with secondary antibodies were similar with IHC. Briefly speaking, tissue sections were incubated with CD3 and Goat Anti-Rabbit IgG (HRP), respectively. After CY3 staining, antigen retrieval and blocking, the first primary antibody was finished. Then tissue sections were incubated with CD20 and Alexa Fluor®647 goat anti-rabbit IgG (H+L), respectively. DAPI and anti-fluorescence quenching agents was used to dye nuclei and for storage. Tissue sections were scanned by 3DHistech (Pannoramic MIDI, Hungary) or observed with fluorescence microscope (Olympus#BX53, Japan).

Lentiviral infection

We constructed overexpressed CCL15 lentivirus named PGMLV-CMV-H_CCL15-3×Flag-EF1-mCherry-T2A-Puro. For lentiviral infection, lentivirus vectors were directly added to Huh7 cells inoculated in 6-well plates. After cells were cultured for 48 hours, the medium was replaced and the transfection

efficiency was evaluated. Cells with lentivirus required 2 ug/ml puromycin treatments for 2 weeks, and then were used for further analyses.

Transwell migration assay

5×10^4 Cells were seeded in the upper chamber of transwell inserts (Corning, USA), which was lay upon the 24-well plates. PMA simultaneously was added into the upper chamber and an appropriate concentration of CCL15 was added into the lower chamber. Cells were cultured for 48 hours and then evaluated the migration proportions using the 20× microscope after cells were fixed by paraformaldehyde and stained by crystal violet (Beyotime, China).

Single-cell suspension preparation and flow cytometric analysis

Adherent macrophages models were digested into cell suspensions by trypsin and subcutaneous tumor tissue were treated with enzyme-assisted microdissection method by collagenase IV (Worthington, USA). Cultured cells or single cells from isolated tumor tissues were suspended in the dilution buffer. The solutions were incubated with primary antibodies at 4℃ and secondary antibodies at room temperature or direct labeled antibodies at 4 ℃ for 30 minutes, respectively. The details and usage of antibodies were shown on Table S7. Cells finally were detected by Flow Cytometer (CyAn ADP Analyzer, Beckman Coulter).

Construction of macrophage model in vitro

THP-1 and U937 were most commonly used cell lines to construct the macrophage model in vitro [1]. Generally, cells were inoculated in 6-well plates

at suitable density. Phorbol 12-myristate 13-acetate (PMA) was added to the medium at 100 ng/μl and cultured for 48 hours. Suspension cell adherence and morphological changes can be used as signs of successful construction of macrophage model. The optimum concentration of CCL15 was determined by pre-experiment. THP-1 and U937 cells stimulated by CCL15 at appropriate concentration were cultured for a week and then collected for further studies.

Animal studies

To further study the recruitment functions of CCL15 in vivo, we implanted 5×10^6 overexpressing CCL15 and control Huh7 cells into the armpit of 8 eight-week-old nude mice respectively and evaluated the tumor growth in 4 weeks later. The resected xenograft was stored in FFPE for further studies.

To comprehensively observe the therapeutical effects of CCL19 and CCL21, we also constructed adeno-associated virus (AAV-8) vectors of overexpressing Ccl19, Ccl21 and Ccl19/Ccl21a as well as control AAVs, respectively. The pre-experimental assays were classified into 4 groups and 1×10^7 cells were injected into one side of armpit of 7 eight-week-old C57/BL6 mice in the subcutaneous xenograft for evaluating the therapeutic effects of 4 groups of AAVs. The formal experiment procedures were shown in the figure (Figure S5E). Briefly speaking, 1×10^7 hepa1-6 cells were first injected into each of the bilateral armpits of 24 eight-week-old mice in the subcutaneous xenograft model. The growth and the largest diameters of emerging tumors were dynamically monitored by vernier caliper per four days, and the volume of the tumor was calculated by the formula:

volume = length*width^2*π/6. Ccl19/Ccl21a-overexpressing AAV (oe-AAV) and control AAV were then intratumorally injected when the largest diameter of the xenograft reached 5 mm. Mice were sacrificed until the tumors grew for 4 weeks and the tumor xenografts were firstly isolated, weighted, photographed and stored in -80℃. Even some tumors were then separated into single cell suspension or fixed in formalin for further studies. In DEN-CCl_4 animal model, two-week-old mice were intraperitoneally injected with 25 mg/kg DEN only once and then 0.5ml/kg CCl_4 once a week, of which the treatments lasted for 14 weeks. After liver tumorigenesis was determined, mice were tail vein injected with oe-AAV and control AAV, and were sacrificed in three months. The tumors and livers were isolated and photographed for further studies.

Statistical analysis

All statistical analyses were performed using GraphPad Prism 8.0.1. Results were presented with mean ± standard error of mean (SEM) for at least three independent experiments. For comparisons, student's t test, paired t test, variance analysis, Wilcoxon signed-rank test and chi-squared test were performed as appropriate. Kaplan-Meier analysis and log-rank test were used to calculate the difference of OS or DFS between various groups. Pearson's correlation test was used to evaluate the p values and correlation coefficients of correlation analyses. receiver operating characteristic curve (ROC) analysis was used to evaluate the predictive values of different expression groups. Online GEPIA2 was used to evaluate the gene expression and survivals of

targets in the TCGA database [2], while TIMER was used to explore the correlations between gene expression and the infiltration of immune cells [3]. Statistical significance was indicated by p value ($p < 0.05$). *p <0.05, **p <0.01, *** p <0.001, ****p <0.0001.

References

1. Hoffmann D, Pilotte L, Stroobant V, Van den Eynde BJ. Induction of tryptophan 2,3-dioxygenase expression in human monocytic leukemia/lymphoma cell lines THP-1 and U937. Int J Tryptophan Res. 2019; 12: 1178646919891736.
2. Tang Z, Kang B, Li C, Chen T, Zhang Z. GEPIA2: an enhanced web server for large-scale expression profiling and interactive analysis. Nucleic Acids Res. 2019; 47: W556-W60.
3. Li T, Fu J, Zeng Z, Cohen D, Li J, Chen Q, et al. TIMER2.0 for analysis of tumor-infiltrating immune cells. Nucleic Acids Res. 2020; 48: W509-W14.

Supplementary Figures and Figure Legends

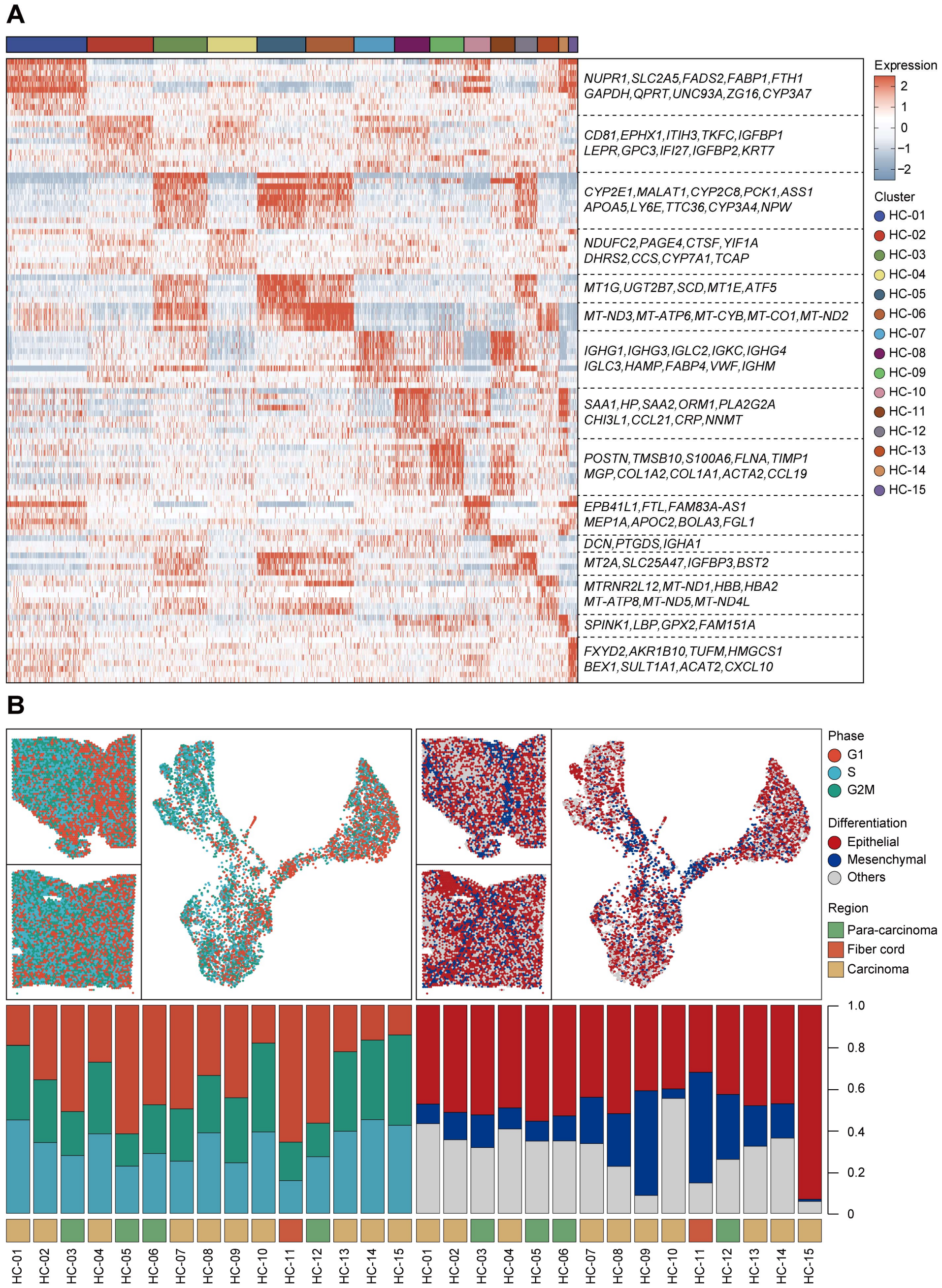

Figure S1. General profiles of 15 clusters from spatial transcriptomics in HCC. A. Heatmap displaying significantly expressed genes of each cluster (logFC≥0.25, p<0.05). **B.** UMAP plot and percentage plot showing the

distribution of cell cycle stages and differentiation origins of all the clusters among three samples, colored by phases, differentiation, and regions.

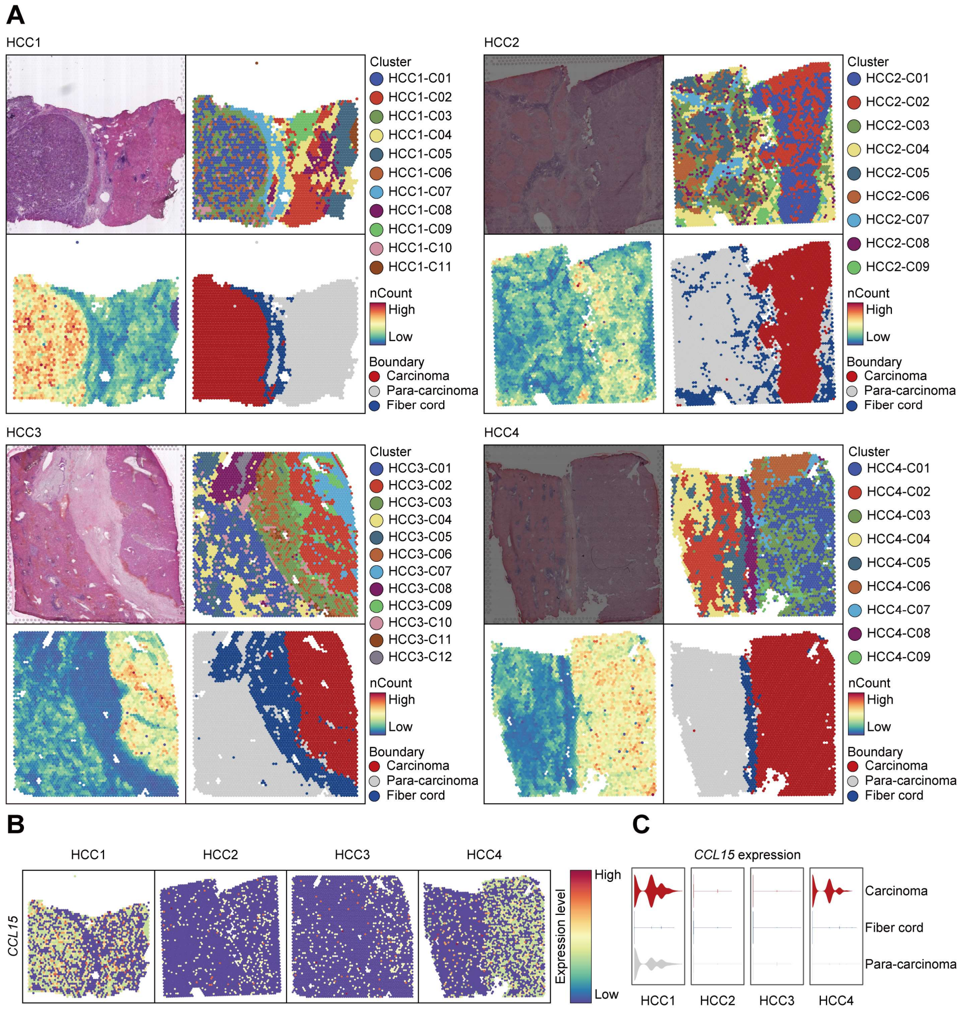

Figure S2. Overall landscapes and spatial features of *CCL15* among 4 pieces of HCC samples from previous ST sequencing data. A. Spatial distribution of clusters and nCounts as well as histological assessment and boundary among 4 pieces of HCC samples (HCC1, HCC2, HCC3, and HCC4). **B-C.** Spatial features (**B**) and relative quantitative analysis (**C**) of *CCL15* among carcinoma, para-carcinoma and fiber cord sectors in four samples.

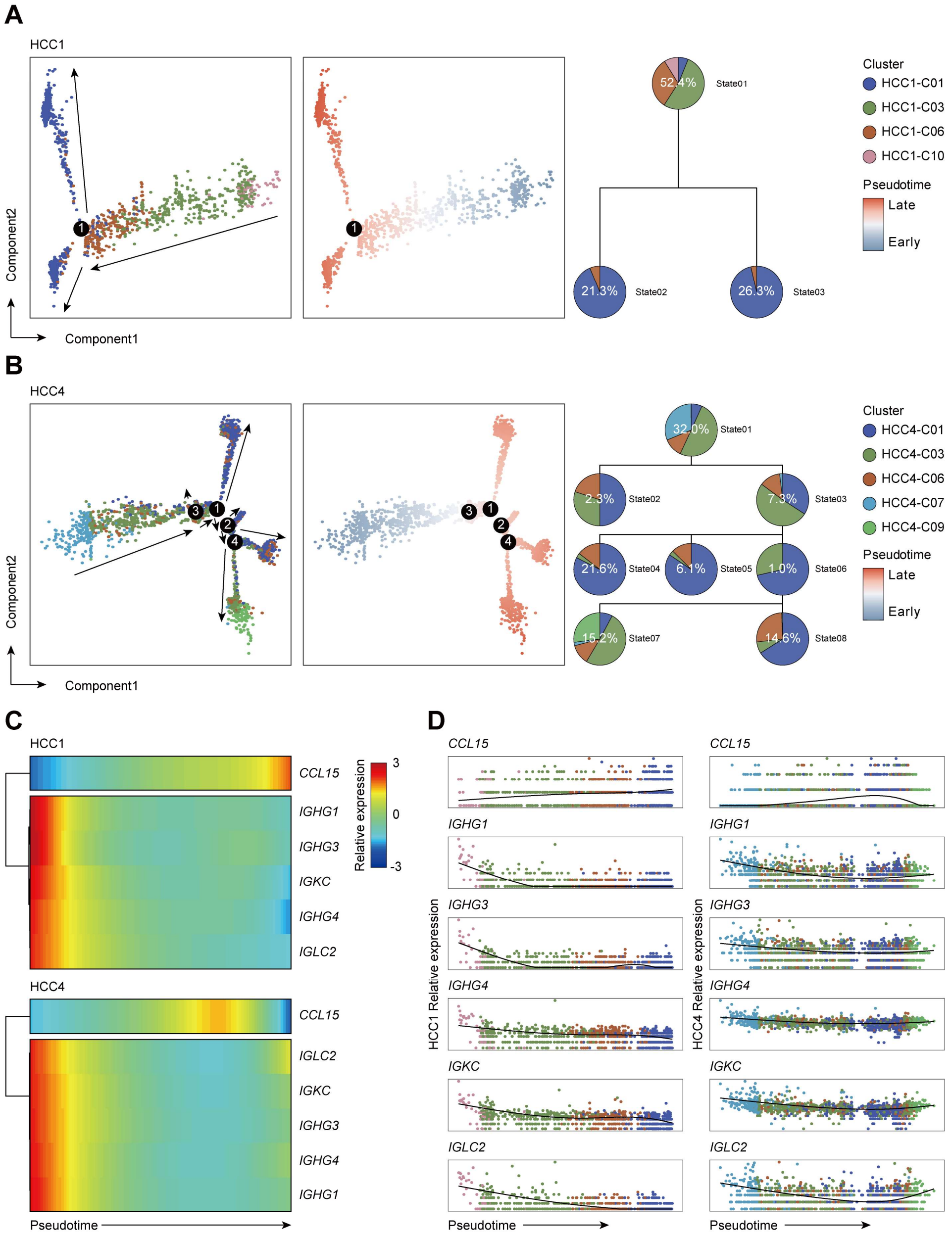

Figure S3. Developmental trajectory and expression trends of specific marker genes such as *CCL15* among HCC1 and HCC4. A-B. Pseudo-time analysis and pie plots showing the developmental trajectory of spots from HCC1 (**A**) and HCC4 (**B**) colored by the clusters, states and pseudo-time. **C.** Heatmap displaying expression changes of specific marker genes in HCC1 and

HCC4 along the pseudo-time trajectory. **D.** Scatter plots and fitting curves presenting the expression trend of selected marker genes among HCC1 and HCC4 along the pseudo-time trajectory.

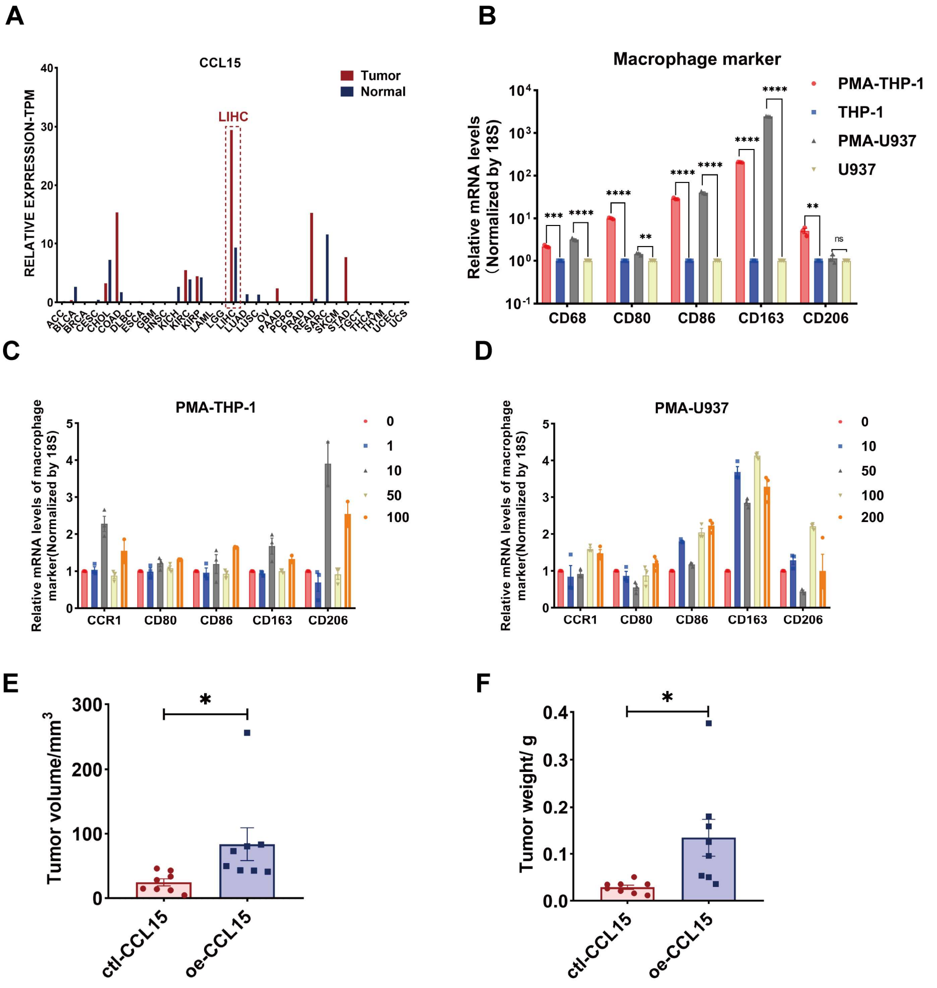

Figure S4. Constructions and marker changes of macrophage model in vitro after CCL15 stimulation. A. Relative expression of *CCL15* among 21 solid tumors among TCGA database (dotted red box represents LIHC). **B.** Expression of macrophage markers after PMA stimulation in THP-1 and U937 cells. **C.** Expression of M1 and M2 markers as well as *CCR1* after various concentration gradients stimulation of CCL15 in PMA-THP-1 macrophage models. **D.** Expression of marker genes after various concentration gradients stimulation of CCL15 in PMA-U937 macrophage models. **E-F.** The tumor

volume (**E**) and tumor weight (**F**) of oe-CCL15 and control group after Huh7 cells were subcutaneously implanted for 4 weeks.

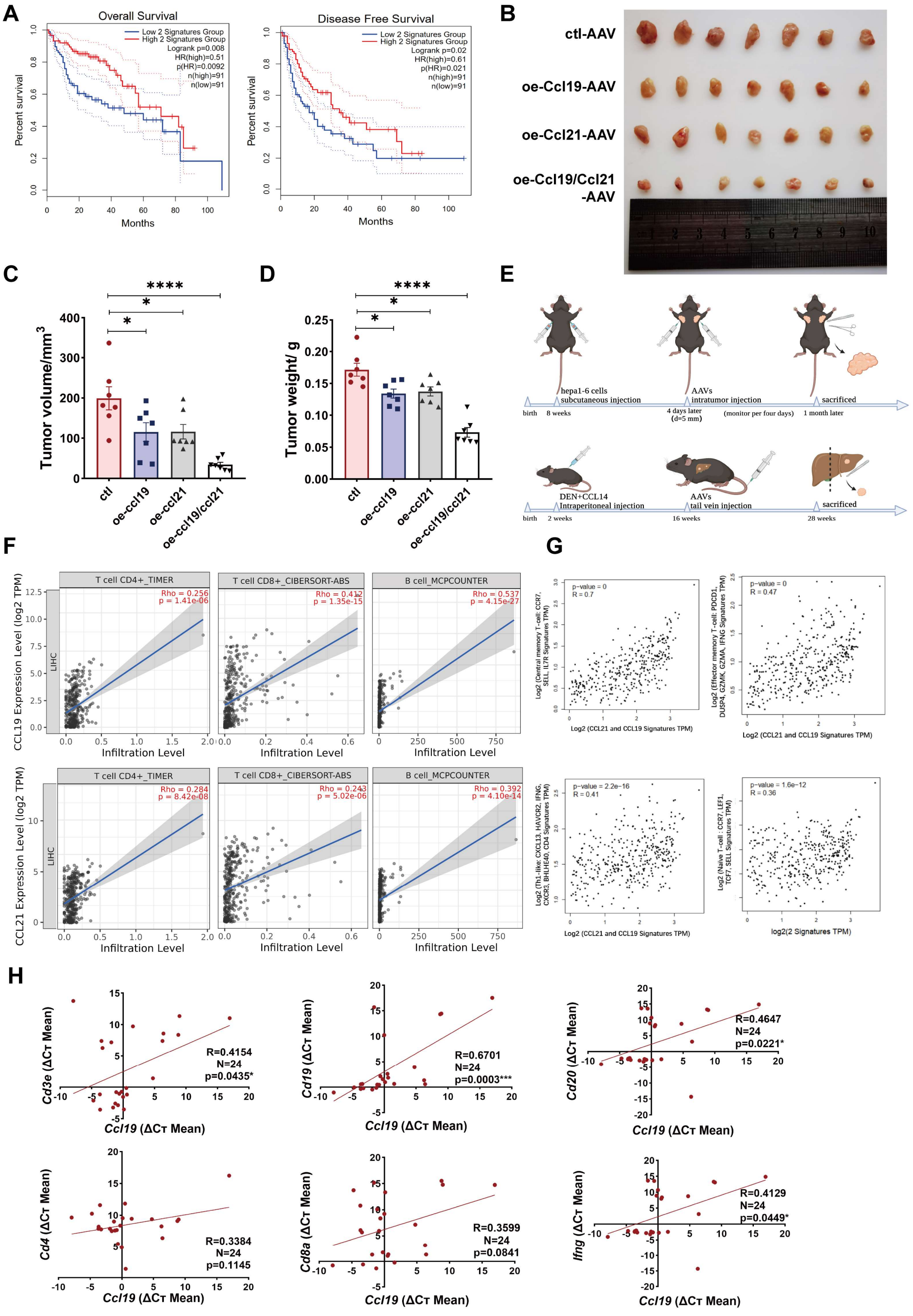

Figure S5. CCL19 and CCL21 inhibit the growth of HCC in two animal models. A. Kaplan-Meier survival curves showing combined predictive role of

CCL19 and CCL21 in OS (top, p=0.008) or RFS (bottom, p=0.02) of HCC patients from TCGA database. **B.** Representative image of subcutaneous xenografts resected from the oe-CCL19 AAV, oe-CCL21 AAV, oe-CCL19/CCL21 AAV and control group (n=7, respectively). **C-D.** Tumor volume (**C**) and (**D**) tumor weight of subcutaneous xenografts after hepa1-6 cell clones were implanted with AAV treatments in 4 groups. **E.** Timelines and procedures of constructing mouse models and AAV treatment. **F.** Correlations between expression of CCL19 and CCL21 and infiltration of CD4+ T cells, CD8+ T cells and B cells in HCC from the TIMER database. **G.** Correlations between expression of combined CCL19 and CCL21 and partial T cell subset markers (several markers combined to indicate cell subsets; R>0.35 are shown). **H.** Correlations between expression of Ccl19 and T or B cell subset markers in subcutaneous tumor xenografts.

Supplementary Tables

Table S1. Clinical Characteristics of 89 HCC patients in CH cohort.

Variable	Number	Number
Age (years, >50; ≤50)	70	19
Gender (male/female)	39	50
γ-GT (u/L, >50; ≤50)	41	48
Albumin/Globulin (A/G, >1.5; ≤1.5)	32	57
AFP (μg/L, >20; ≤20)	53	36
HBsAg (positive/negative)	63	24
HBeAg (positive/negative)	14	75
Edmonson’s grade (Ш+Ⅳ; Ⅰ+Ⅱ)	11	78
Tumor size (cm, >5; ≤5)	58	41
Tumor number (multiple/solitary)	82	7
Encapsulation (present/absent)	21	68
Tumor thrombus (present/absent)	29	60
BCLC stage (B+C; A)	67	22
All cases	89	

Table S2. Signatures for cell cycle phases based on annotations.

G2M phases signature genes		S phase signature genes	
HMGB2	*KIF23*	*MCM5*	*RFC2*
CDK1	*HMMR*	*PCNA*	*RPA2*
NUSAP1	*AURKA*	*TYMS*	*NASP*
UBE2C	*PSRC1*	*FEN1*	*RAD51AP1*
BIRC5	*ANLN*	*MCM2*	*GMNN*
TPX2	*LBR*	*MCM4*	*WDR76*
TOP2A	*CKAP5*	*RRM1*	*SLBP*
NDC80	*CENPE*	*UNG*	*CCNE2*
CKS2	*CTCF*	*GINS2*	*UBR7*
NUF2	*NEK2*	*MCM6*	*POLD3*
CKS1B	*G2E3*	*CDCA7*	*MSH2*
MKI67	*GAS2L3*	*DTL*	*ATAD2*
TMPO	*CBX5*	*PRIM1*	*RAD51*
CENPF	*CENPA*	*UHRF1*	*RRM2*
TACC3	*KIF20B*	*MLF1IP*	*CDC45*
FAM64A	*HJURP*	*HELLS*	*CDC6*
SMC4	*CDCA3*	*CLSPN*	*EXO1*
CCNB2	*HN1*	*POLA1*	*TIPIN*
CKAP2L	*CDC20*	*CHAF1B*	*DSCC1*
CKAP2	*TTK*	*BRIP1*	*BLM*
AURKB	*CDC25C*	*E2F8*	*CASP8AP2*
BUB1	*KIF2C*	*CLSPN*	*USP1*
GTSE1	*CDCA2*		
ANP32E	*NCAPD2*		
KIF11	*RANGAP1*		
TUBB4B	*DLGAP5*		
ECT2	*CDCA8*		

Table S3. Signature genes for epithelial and mesenchymal differentiation.

Epithelium marker genes		EMT marker genes
EPCAM	*KLK10*	*VIM*
KRT16	*KLK11*	*DPDN*
KRT6A	*KRT17*	*ITGA5*
KRT6B	*KRT75*	*ITGA6*
KRT6C	*S100A7*	*TGFBI*
CLDN4	*S100A8*	*LAMC2*
KLK5	*S100A9*	*MMP1*
KLK6	*CLDN1*	*MMP2*
KLK7	*CLDN7*	*MMP3*
KLK8	*SPRRIB*	
KLK9		

Table S4. Cell markers for immune cells.

T cell feature genes	B cell feature genes	NK cell feature genes	Myeloid cell feature genes
CD3D	*CD19*	*NCAM1*	*ITGAX*
CD3E	*MS4A1*	*KLRF1*	*CD33*
CD3G	*CD79A*	*NCR1*	*CEACAM8*
		KLRC1	*CD68*
			CD163
			LYZ

Table S5. Correlations between clinical characteristics of 61 HCC patients and CCL15 & CD163 expression level.

Variable	*CCL15 & CD163*#		*p* value
	High	Low	
Age (years, >50; ≤50)	29:6	21:5	0.8339
Gender (male/female)	15:20	11:15	0.9658
γ-GT (u/L, >50; ≤50)	19:16	13:13	0.7403
Albumin/Globulin (A/G, >1.5; ≤1.5)	25:10	17:9	0.6142
AFP (μg/L, >20; ≤20)	23:12	13:13	0.2172
HBsAg (positive/negative)	22:13	21:5	0.1293
HBeAg (positive/negative)	7:28	5:21	0.9404
Edmonson's grade (Ш+Ⅳ; Ⅰ+Ⅱ)	5:30	1:25	0.1869
Tumor size (cm, >5; ≤5)	13:22	6:20	0.2407
Tumor number (multiple/solitary)	1:34	2:24	0.3878
Encapsulation (present/absent)	4:31	8:18	0.0602
Tumor thrombus (present/absent)	13:22	8:18	0.6044
BCLC stage (B+C; A)	30:5	19:7	0.2195
All cases	35	26	/

#According to the cutoff of CCL15 and CD163 using X-tile software, the table showed the correlations between clinical characteristics and $CCL15^{hi}CD163^{hi}$ or $CCL15^{lo}CD163^{lo}$ groups, chi-square test was used to test the statistical significance, n=61, $p < 0.05$ as the cutoff values.

Table S6. Sequences of primers for quantitative real-time PCR.

Primer	Sequences (5'- 3')
hum-CD80-F	AAACTCGCATCTACTGGCAAA
hum-CD80-R	GGTTCTTGTACTCGGGCCATA
hum-CD86-F	CTGCTCATCTATACACGGTTACC
hum-CD86-R	GGAAACGTCGTACAGTTCTGTG
hum-CD163-F	TTTGTCAACTTGAGTCCCTTCAC
hum-CD163-R	TCCCGCTACACTTGTTTTCAC
hum-CD206-F	CGAGGAAGAGGTTCGGTTCACC
hum-CD206-R	GCAATCCCGGTTCTCATGGC
mus-Cd19-F	CTTGGTATCGAGGTAACCAGTCA
mus-Cd19-R	ACAATCACTAGCAAGATGCCC
mus-Cd8A-F	CCGTTGACCCGCTTTCTGT
mus-Cd8A-R	TTCGGCGTCCATTTTCTTTGG
mus-Ifng-F	ATGAACGCTACACACTGCATC
mus-Ifng-R	CCATCCTTTTGCCAGTTCCTC
mus-Cd4-F	CTAGCTGTCACTCAAGGGAAGA
mus-Cd4-R	CGAAGGCGAACCTCCTCTAA
mus-Cd3e-F	TCAGCCTCCTAGCTGTTGG
mus-Cd3e-R	GTCAACTCTACACTGGTTCCTG
mus-Cd20-F	AACCTGCTCCAAAAGTGAACC
mus-Cd20-R	CCCAGGGTAATATGGAAGAGGC
mus-Ccl19-F	GGGGTGCTAATGATGCGGAA
mus-Ccl19-R	CCTTAGTGTGGTGAACACAACA

mus-Actb-F	GGCTGTATTCCCCTCCATCG
mus-Actb-R	CCAGTTGGTAACAATGCCATGT
hum-18S-F	GGAGAGGGAGCCTGAGAAACG
hum-18S-R	TTACAGGGCCTCGAAAGAGTCC

Table S7. The details of antibodies used in the study.

Antibody names	Brand	Product code	Usage
Anti-CCL15	Abcam	Ab221040	IHC, 1:100<br>WB, 1:1000
Anti-CD163	Abcam	Ab181422	IHC, 1:250<br>FC, 1:60
Goat anti-rabbit IgG HRP	Abcam	Ab205718	IHC, 1:2000
Anti-CD206	Proteintech	60143-1-Ig	FC, 1:20
Anti-CD3	Abcam	Ab16669	FC, 1:20<br>IF, 1:500<br>WB, 1:40
Anti-CD20	Abcam	Ab64088	FC, 1:20<br>IF, 1:100<br>WB, 1:40
Anti-β-actin	Servicebio	GB15001	WB, 1:1000
IRdye680 Goat anti-mouse IgG	Licor	926-32220	WB, 1:10000
IRdye800 Goat anti-rabbit IgG	Licor	926-32211	WB, 1:10000
Goat Anti-Mouse IgG, FITC	Proteintech	SA00003-1	FC, 1:20
Goat Anti-Rabbit IgG, R-PE	Proteintech	SA00008-2	FC, 1:20
Mouse IgG2a FITC Isotype	Proteintech	FITC-65208	FC, 1:20
Armenian Hamster IgG PE Isotype	Proteintech	PE-65210	FC, 1:20
